# Youth Labour Market Participation in Africa: A Cross-National Analysis of Employment, Unemployment, Sectoral Distribution, and Educational Attainment Among the 15–35 Age Cohort, 2026

**DOI:** 10.12688/f1000research.179407.1

**Published:** 2026-04-22

**Authors:** Said Maizzou, Abdelhak Anajar, Mohamed Rahmo

**Affiliations:** 1 Laboratory LASMO, Universite Hassan 1er, Settat, casablanca-Settat, 26000, Morocco

**Keywords:** youth unemployment; NEET; Africa; labour force partic-ipation; sectoral employment; educational attainment; gender gap; ILO modelled estimates

## Abstract

**Background:**

Africa hosts the world’s largest youth cohort, yet its labour markets remain characterised by structural imbalances, agricultural dependence, gender inequalities, and educational credential mismatches. This study provides a comprehensive, cross-national analysis of youth labour market participation across all 53 African Union member states in 2026, disaggregated by sex, age group, economic sector, and educational attainment.

**Methods:**

A cross-sectional descriptive-correlational analysis was conducted using International Labour Organisation ( *ILO*) harmonised modelled estimates for the 15–35 age cohort. Youth unemployment rates ( *YUR*), labour force participation rates ( *LFPR*), and notinemployment,educationortraining ( *NEET*) rates were computed by country, sub-region, sex, age group, and education level. Pearson’s correlation coefficient tested the association between *YUR* and *NEET* rate at country level.

**Results:**

Of 545.1 million African youth aged 15–35, 311.5 million (57.1%) were employed, 23.1 million (4.2%) unemployed, 81.7 million (15.0%) inactive, and 128.8 million (23.6%) in education. The continental *YUR* was 6.91%; North Africa recorded 11.09% versus 6.40% in sub-Saharan Africa. Country-level *YUR* ranged from 0.4% (Niger) to 37.4% (Eswatini). The *NEET* rate reached 19.23% (104.8 million youth). Agriculture dominated employment (45.9%). Female *YUR*(7.56%) exceeded male *YUR*(6.40%). Upper secondary and tertiary graduates accounted for 52.7% of unemployment. A strong positive correlation existed between *YUR* and *NEET* rate ( *r* = 0.720, *p* = *<* 0.001).

**Conclusions:**

African youth labour markets exhibit pronounced heterogeneity, structural informality, and credential–employment mismatches. Targeted policies addressing structural transformation, skills alignment, gender equity, and social protection are urgently required.

## Introduction

### Background and rationale

Africa is undergoing an unprecedented demographic transition. By 2026, the continent hosts approximately 1.5 billion people, of whom an estimated 545 million are young persons aged 15–35 the largest youth cohort in the world. This demographic trajectory presents a dual opportunity: if adequately absorbed into productive employment, Africa’s youth could generate a demographic dividend capable of sustaining economic growth for decades; conversely, failure to integrate young people into the labour market risks perpetuating cycles of poverty, social exclusion, and political instability. ^1, 2, 3, 4, 5^

Despite economic growth averaging 4–5% per annum across sub-Saharan Africa since 2000, employment generation has consistently lagged behind labour-force expansion. A structural disconnect persists between the sectors absorbing the largest share of workers predominantly sub-sistence agriculture and the informal economy and the productive, high-value activities needed to deliver sustain-able income gains. ^6, 7^ Simultaneous rapid expansion of tertiary enrolment without commensurate growth in high-skilled employment has generated widespread credential–employment mismatch. ^8, 9, 10^

Gender represents a critical axis of labour market disadvan-tage. Young women face lower participation rates, higher unemployment, and greater concentration in precarious informal work relative to male counterparts, reflecting entrenched social norms, discriminatory hiring, and disproportionate care burdens. ^11, 12^ The *NEET* indicator has emerged as a more comprehensive proxy for youth disengagement than unemployment alone, capturing both active job-seekers and discouraged workers who have with-drawn from the labour market entirely. ^13, 14, 15^

### Research questions

This study is guided by four research questions ( **RQ**):

**RQ**_1_ What is the distribution of labour market status em-ployed, unemployed, inactive, in education/training among African youth aged 15–35 across all 53 coun-tries in 2026?

**RQ**_2_ To what extent do *YUR*, *NEET* rates, and *LFPR* vary by country, sub-region, age group, and sex?

**RQ**_3_ What is the relationship between educational attain-ment and youth unemployment, and is there evidence of credential–employment mismatch?

**RQ**_4_ How does the sectoral distribution of youth employ-ment differ by sex, and what structural implications does this hold for gender equity?

### Hypotheses

Building on theory and recent empirical evidence, ^16, 17, 18, 19^ this study tests four hypotheses ( **H**):

**H** _1_
*YUR* is significantly higher in North Africa than in sub-Saharan Africa, reflecting formal-sector scarcity and widespread informal agricultural employment.

**H** _2_
Female youth face systematically higher *YUR* and lower *LFPR* than male youth.

**H** _3_
Youth with upper secondary and tertiary qualifications account for a disproportionate share of unemploy-ment relative to their population weight (credential–employment mismatch).

**H** _4_
*YUR* and NEET rate are positively correlated at country level.

### Significance and scope

Pan-African comparative analyses simultaneously exam-ining *YUR*, *NEET*, *LFPR*, sectoral structure, and educational attainment with full sex disaggregation across all 53 African countries remain scarce. ^16, 1^ This study fills that gap and provides an evidence base for policies aligned with the African Union’s Agenda 2063 and United Nations Sustainable Development Goal 8 (Decent Work and Economic Growth).

## Methods

### Study design

A cross-sectional, descriptive-correlational study design was employed. Analysis was conducted at country level across all 53 African Union member states for the calendar year 2026. The study follows the STROBE (Strengthening the Reporting of Observational Studies in Epidemiology) guidelines, adapted for secondary aggregate data. ^20^ Geospatial analysis follows Bayesian approaches that account for spatial dependence and heterogeneity across countries and sub-regions. ^21^

### Data source

All data derive from *ILO* harmonised modelled labour market estimates (ILOSTAT, 2024 release). ^22^ These estimates are produced through a multi-step framework com-bining national household survey microdata, administra-tive records, and econometric imputation for countries with incomplete or outdated survey coverage, ensuring cross-national comparability. ^23^ The target population is youth aged 15–35 years, consistent with the African Union’s definition of youth.

Four labour-market status variables were examined:

• **Employed**: performed *≥*1 hour of paid or self-employed work in the reference period; disaggregated by country, single year of age, sex, and ISIC Rev.4 sector (aggregated as Agriculture, Industry, Services).
• **Unemployed**: not in employment, actively seeking work, and available to start; disaggregated by country, age, sex, and educational attainment (no education; primary; lower secondary; upper secondary; tertiary).
• **Inactive**: neither employed nor seeking work (including discouraged workers); disaggregated analogously.
• **Students**: currently enrolled in formal or non-formal education or training; disaggregated analogously.

### Outcome indicators

Three primary indicators were computed for each country:

$$\mathrm{YUR}=\frac{\text{Unemployed Employed}}{\text{Employed}+\text{Unemployed}}\times 100$$ (1)

$$\text{LFPR}=\frac{\text{Employed}+\text{Unemployed}}{\text{TotalYouth}}\times 100$$ (2)

$$\text{NEET}=\frac{\text{Unemployed}+\text{Inactive}}{\text{TotalYouth}}\times 100$$ (3)

Definitions conform to *ILO* resolutions adopted at the 13th and 19th International Conferences of Labour Statisticians. ^23^

Sub-regional aggregates were computed for North Africa (Algeria, Egypt, Libya, Morocco, Sudan, Tunisia) and sub-Saharan Africa (the remaining 47 countries).

### Statistical analysis

All analyses were performed in Python 3.12 using *pan-das* v2.2 and *NumPy* v1.26; figures were produced with *matplotlib* v3.10. To test H _4_, Pearson’s correlation coefficient *r* was computed between country-level *YUR* and *NEET* rate ( *n* = 53; significance threshold *α* = 0.05). Cross-country inequality in *YUR* was quantified using the Gini coefficient. All descriptive statistics are reported to one decimal place.

### Ethical considerations

This study uses exclusively publicly available, aggregate, anonymized *ILO* modelled estimates. No primary data collection involving human participants or animals was undertaken. Formal ethical approval was not required under applicable institutional guidelines. The dataset is freely accessible at https://africayouthjobs.io.

## Results

### 3.1 Overall labour market status distribution (RQ _1_)

**Figure 1.**
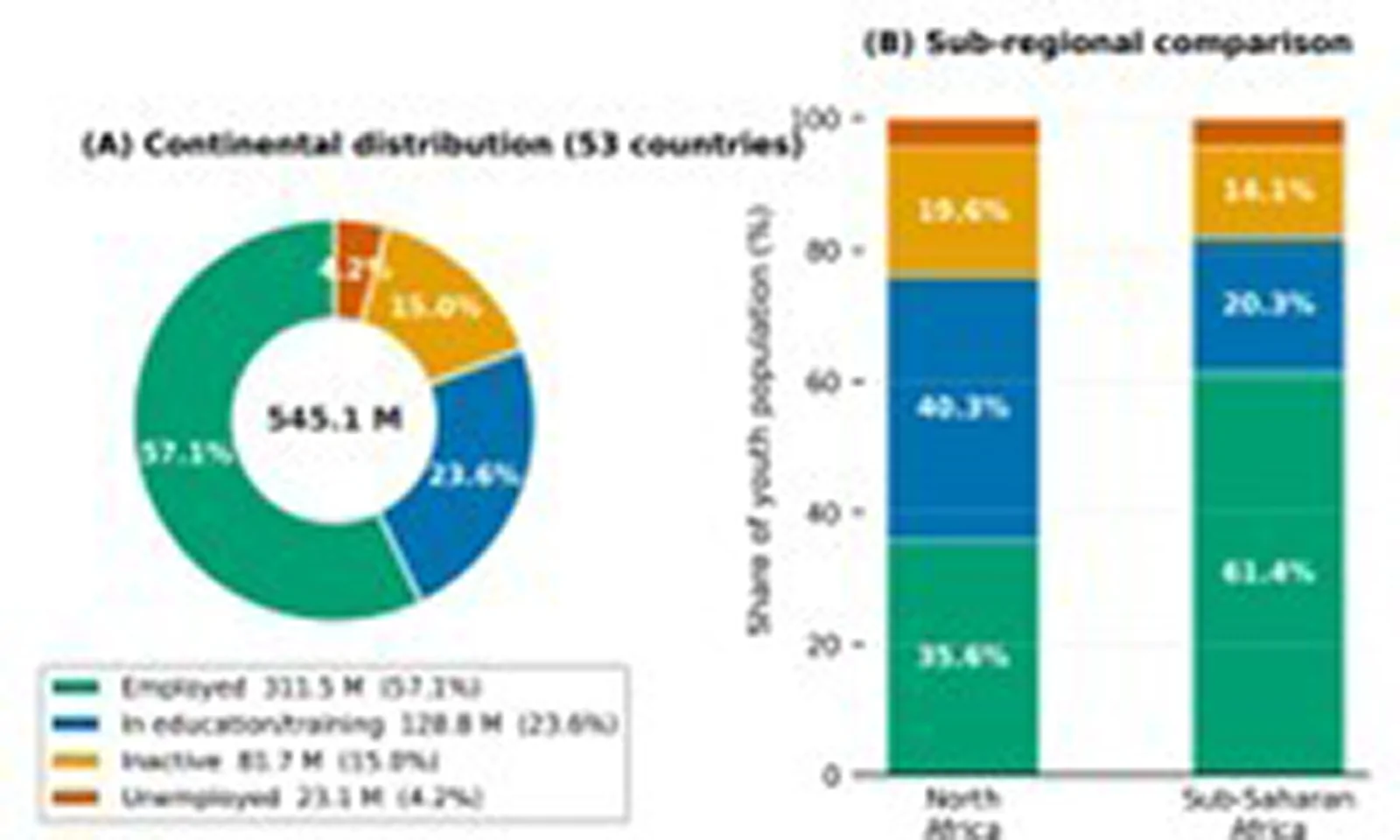
Distribution of labour market status among african youth (15–35 Years), 2026. Panel (A): proportional distribution across four statuses for all 53 coun-tries combined (total = 545.1 million). Panel (B): sub-regional comparison between North Africa ( *n* = 6) and Sub-Saharan Africa ( *n* = 47). Source: Author’s calculations based on (World Data Lab 2026).

The total African youth population aged 15–35 comprised an estimated 545.1 million individuals across 53 countries in 2026 ( Figure 1). Of these, 311.5 million (57.1%) were employed, 23.1 million (4.2%) were unemployed, 81.7 million (15.0%) were economically inactive, and 128.8 million (23.6%) were in education or training. The continental *YUR* was 6.91% and the *LFPR* was 61.38%. The *NEET* rate reached 19.23%, representing approximately 104.8 million youth neither employed nor in education/training a population at elevated risk of long-term human-capital atrophy. ^13^

### 3.2 Cross-country heterogeneity and sub-regional patterns (RQ _2_; H _1_)

**Figure 2.**
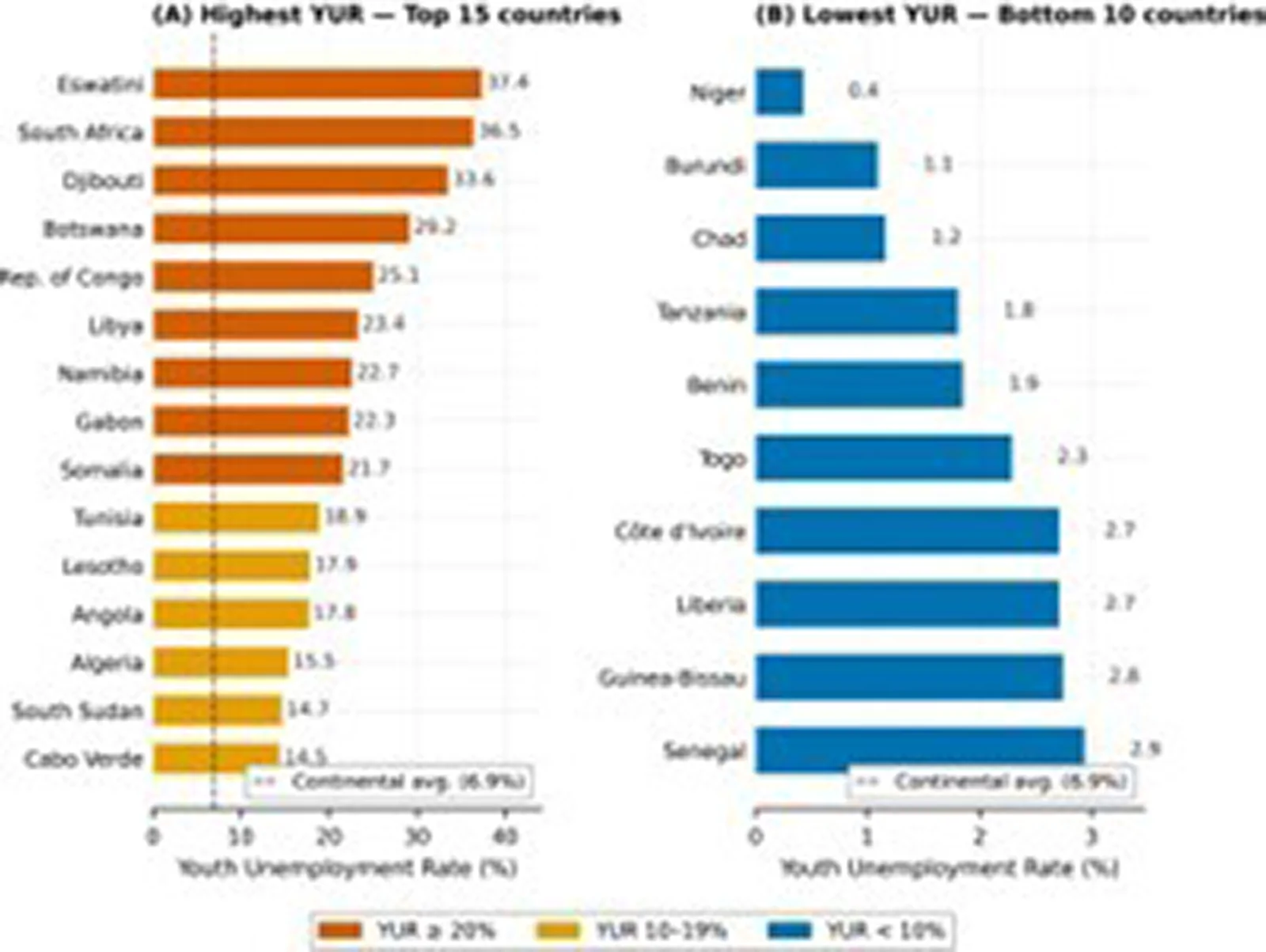
Youth unemployment rate (YUR) by country, Africa, 2026. Panel (A): 15 countries with highest *YUR.* Panel (B): 10 countries with lowest *YUR.* Dashed vertical line = continental average (6.91%). Colour coding: red *≥*20% (critical); amber 10–19% (high); teal *<*10% (moderate/low). Source: Author’s calculations based on (World Data Lab 2026).

Figure 2 illustrates the pronounced cross-country heterogeneity in *YUR.* Rates range from 37.4% in Eswatini to 0.4% in Niger a 93-fold differential. The Gini coefficient for *YUR* across 53 countries equals 0.61, indicating very high cross-country inequality. The 15 highest- *YUR* countries include Eswatini (37.4%), South Africa (36.5%), Djibouti (33.6%), Botswana (29.2%), and the Republic of the Congo (25.1%), sharing characteristics of structurally constrained formal sectors relative to growing youth labour supply. ^18, 19^

Consistent with **H**_1_, North Africa recorded a substantially higher *YUR*(11.09%) than sub-Saharan Africa (6.40%), reflecting the distinctive characteristics of North African labour markets: large but saturated public sectors, limited private-sector dynamism, and elevated rates of educated unemployment, especially among women. ^24, 17^ Paradoxically, several sub-Saharan African countries record very low *YUR* (Niger 0.4%, Burundi 1.1%) yet high *NEET* rates (13.9% and 9.5%, respectively), reflecting necessity employment where the absence of social protec-tion compels any form of work regardless of productivity. ^2, 25^

Table 1 presents the full set of indicators for all 53 countries (selected, ranked by *YUR*).

**Table 1.** Youth labour market indicators by country, Africa, 2026 (selected countries, Ranked by YUR).

Country	YUR (%)	NEET (%)	LFPR (%)	Employed (000 s)	Unemployed (000 s)
Eswatini	37.4	37.3	46.9	139	83
South Africa	36.5	33.6	53.2	7 727	4 434
Djibouti	33.6	40.9	28.1	81	41
Botswana	29.2	31.3	63.2	444	183
Rep. of Congo	25.1	24.9	60.7	1 029	345
Libya	23.4	25.4	41.2	764	233
Namibia	22.7	25.2	51.9	400	117
Gabon	22.3	24.4	43.3	298	85
Somalia	21.7	36.9	28.9	1 505	417
Tunisia	18.9	18.5	44.9	1 329	311
Lesotho	17.9	31.4	53.0	387	84
Angola	17.8	21.0	69.1	7 731	1 671
Algeria	15.5	35.0	40.0	4 696	859
South Sudan	14.7	23.1	69.7	2 347	405
Morocco	11.6	27.0	42.4	4 661	612
Rwanda	12.7	21.9	62.0	2 988	435
Egypt	8.9	20.6	41.0	14 775	1 451
Mozambique	7.9	16.3	75.1	9 047	781
Kenya	7.3	16.6	57.7	11 285	895
Ethiopia	3.8	14.5	66.0	32 179	1 268
Nigeria	3.8	11.1	77.3	62 139	2 463
Uganda	3.1	11.3	78.2	15 192	491
Tanzania	1.8	9.3	80.3	19 996	369
Niger	0.4	13.9	77.4	7 630	33
**Continental total**	**6.9**	**19.2**	**61.4**	**311 499**	**23 114**

Source: Author’s calculations based on (World Data Lab 2026).

### 3.3 Age-group differentials (RQ _2_)

Youth unemployment exhibits a non-linear age profile. Rates were highest in the 20–24 cohort (9.52%) and in the 15–19 cohort (9.41%), declining to 4.83% for ages 25–29 and 5.19% for ages 30–35. This pattern reflects transitional unemployment among recent school-leavers. ^1^ The modest uptick for the 30–35 group is consistent with labour-market scarring, whereby prolonged early unemployment reduces long-run employability. ^13, 26^ Evidence from hysteresis tests confirms that youth unemployment persistence varies significantly across African countries and income levels. ^26^

### 3.4 Gender disparities (RQ _2_, RQ _4_; H _2_)

**Figure 3.**
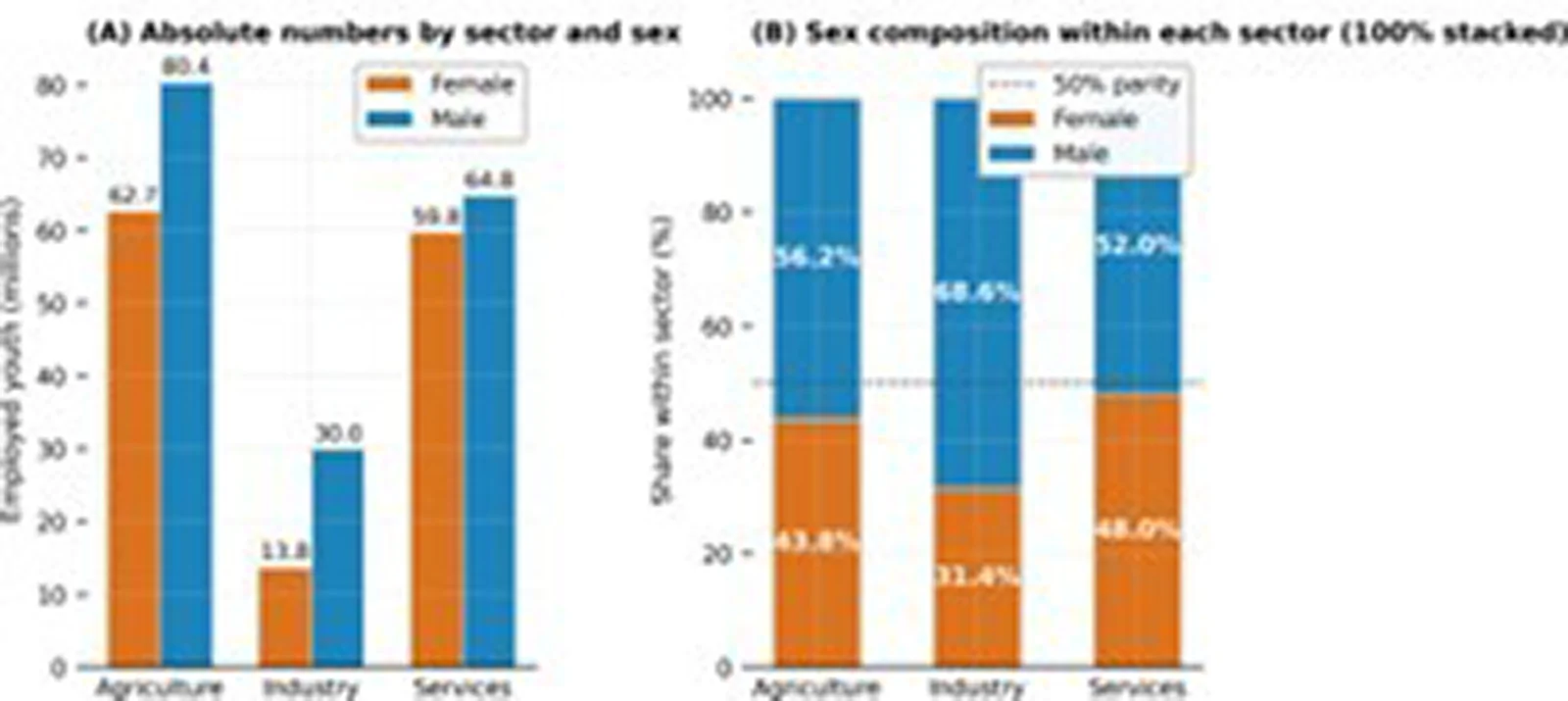
Youth employment by economic sector and sex, Africa, 2026. Panels (A)–(B): proportional sectoral distribution for female and male employed youth. Panel (C): absolute figures by sector and sex (millions). Sectors: Agriculture (green), Industry (amber), Services (navy). Source: Author’s calculations based on (World Data Lab 2026).

Supporting **H**_2_, female *YUR*(7.56%) exceeded male *YUR*(6.40%), a gap of 1.16 percentage points. Female employment totalled 136.3 million versus 175.2 million for males. Gender disparities are further visible in the sectoral distribution ( Figure 3; Table 2). Women were markedly under-represented in industry (10.1% of female employment vs. 17.1% of male employment), whilst services claimed a higher share among women (43.9%) than men (37.0%), reflecting the concentration of women in informal service activities, domestic work, and petty trade. ^11^

**Table 2.** Youth employment by economic sector and sex, Africa, 2026.

Sector	Female (000 s)	Male (000 s)	Total (000 s)	Share of total (%)
Agriculture	62 697	80 423	143 120	45.9
Services	59 789	64 806	124 595	40.0
Industry	13 770	30 014	43 784	14.1
**Total**	**136 256**	**175 243**	**311 499**	**100.0**

Source: Author’s calculations based on (World Data Lab 2026).

### 3.5 Sectoral distribution (RQ _4_)

Agriculture dominated youth employment at 45.9% (143.1 million), followed by services at 40.0% (124.6 million) and industry at 14.1% (43.8 million) (creftab:t2). Agricultural dominance is most pronounced in Niger, Tanzania, and Ethiopia, where low measured *YUR* coexists with extensive subsistence farming. Countries with more diversified structures South Africa, North African states record higher industrial and services shares alongside higher formal unemployment. ^6, 27, 7^ The agriculture sector represents both a repository of last-resort employment and a potential engine for inclusive development if adequately modernised and linked to youth-inclusive value chains. ^28, 29, 30^

### 3.6 Unemployment by educational attainment (RQ _3_; H _3_)

**Figure 4.**
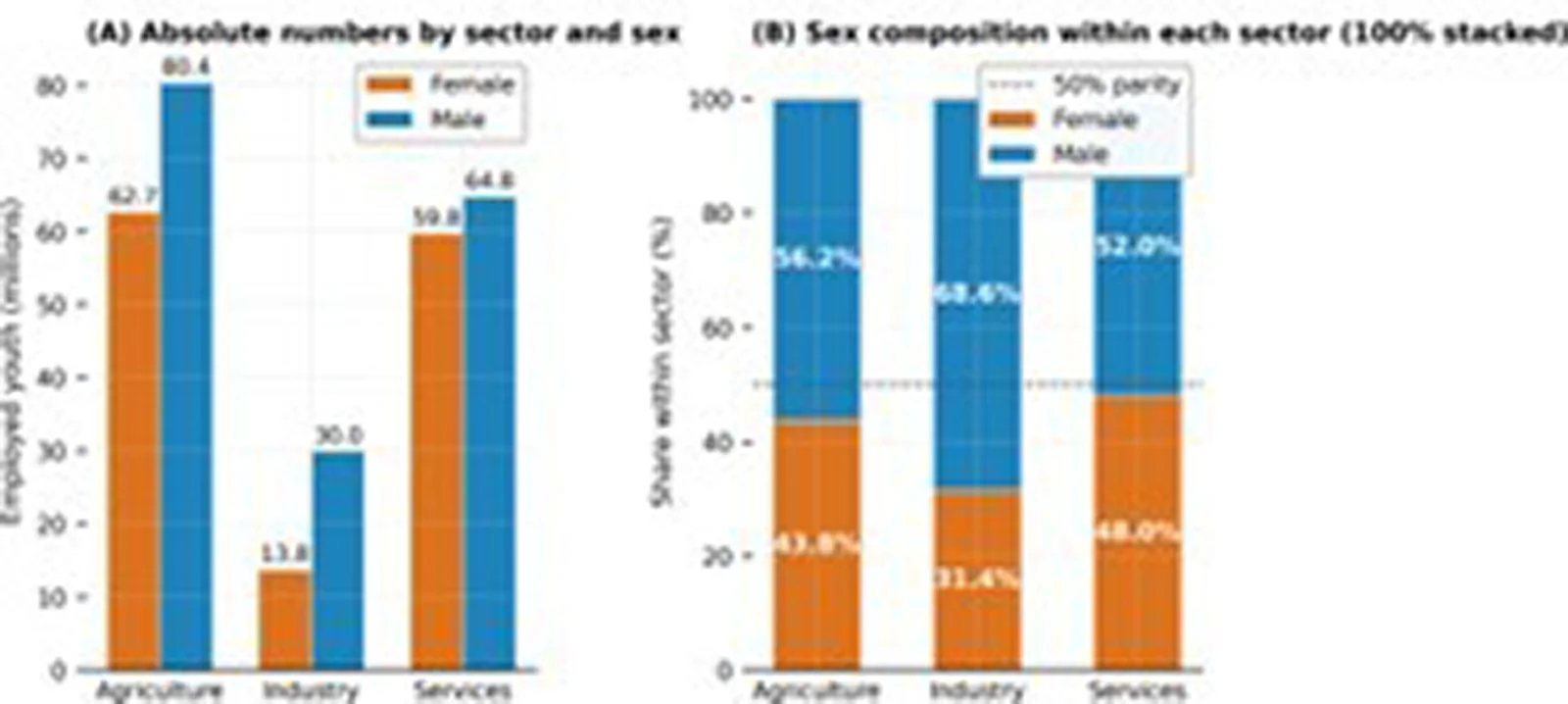
Youth unemployment by educational attainment and sex, Africa, 2026. Panels (A)–(B): proportional sectoral distribution for female and male employed youth. Panel (C): absolute figures by sector and sex (millions). Sectors: Agriculture (green), Industry (amber), Services (navy). Source: Author’s calculations based on (World Data Lab 2026).

Figure 4 and Table 3 strongly support **H**_3_. Upper secondary graduates constituted 32.9% of all unemployed youth (7.6 million) and tertiary graduates 19.8% (4.6 million); together, 52.7% of total unemployment. By contrast, youth without formal education accounted for only 15.7% (3.6 million). This *educated unemployment paradox* reflects structural mismatches between curricula and private-sector demand. ^8, 31, 9, 32^ The persistent skills gap particularly in digital and soft skills constrains even educated youth from transitioning into productive employment. ^33, 34, 35^

**Table 3.** Youth unemployment by educational attainment and sex, Africa, 2026.

Education Level	Female (000 s)	Male (000 s)	Total (000 s)	Share of total (%)
Upper secondary	3 925	3 672	7 597	32.9
Tertiary	2 233	2 340	4 573	19.8
Lower secondary	1 895	2 635	4 530	19.6
No education	1 731	1 900	3 631	15.7
Primary	1 354	1 429	2 783	12.0
**Total**	**11 138**	**11 976**	**23 114**	**100.0**

Source: Author’s calculations based on (World Data Lab 2026).

By sex, women with upper secondary credentials constituted 35.3% of female unemployment (3.9 million) versus 30.7% for males (3.7 million), reinforcing the compounding role of gender discrimination in amplifying educational mismatch. ^11^

### 3.7 YUR–NEET correlation (H _4_)

**Figure 5.**
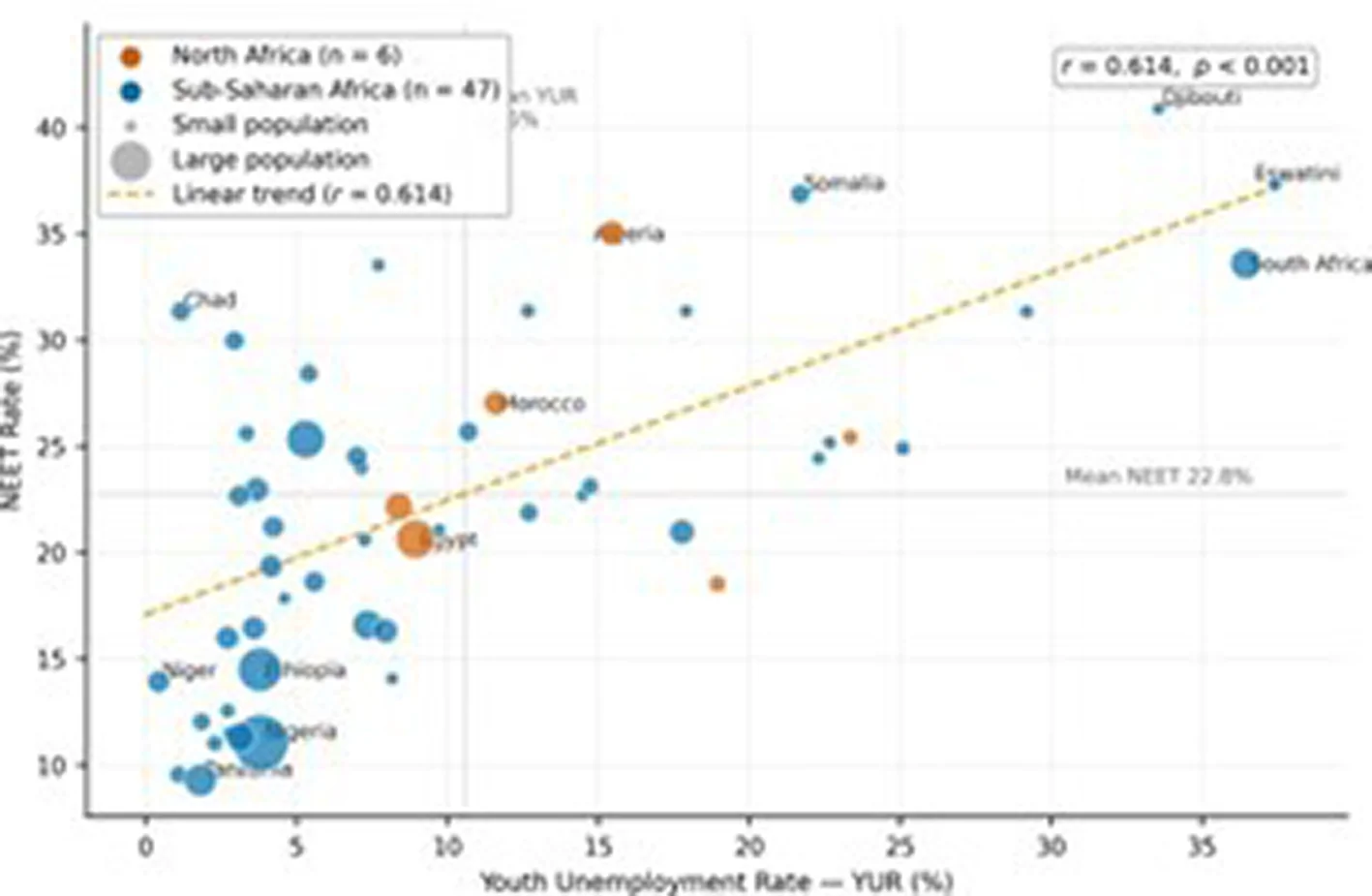
Relationship between youth unemployment rate (YUR) and NEET rate by country, Africa, 2026. Bubble size is proportional to youth population aged 15–35. Red = North Africa; navy = Sub-Saharan Africa. Dotted lines denote continental averages on each axis. Dashed regression line: *r* = 0.720, *p* = *<* 0.001 ( *n* = 53). Source: Author’s calculations based on (World Data Lab 2026).

Pearson’s correlation between country-level *YUR* and *NEET* rate ( *n* = 53) yielded *r* = 0.720 ( *p* = *<* 0.001), confirming **H**_4_ ( Figure 5). A positive association between formal labour-market stress and broader youth disengagement is therefore evident. Notable deviations from the trend include Algeria ( *YUR* 15.5%, *NEET* 35.0%) high on both dimensions and Chad ( *YUR* 1.2%, *NEET* 31.3%) low unemployment yet extreme inactivity reflecting necessity employment in the absence of social protection.

## Discussion

### 4.1 Principal findings in context

This study provides a harmonised, pan-African portrait of youth labour markets in 2026, confirming all four hypotheses. The continental *YUR*of 6.91% (Gini coefficient 0.61) masks a *NEET* rate of 19.23%, a 93-fold country-level *YUR* range, a strong YUR–NEET correlation ( *r* = 0.720), and persistent structural imbalances. ^2, 36^ These findings underscore the inadequacy of *YUR* alone as a policy metric and highlight the need for multi-dimensional frame-works. ^13, 14^ Income inequality has been identified as a key accelerator of youth unemployment across African countries, ^5^ while political instability exerts additional upward pressure through its effects on investment and labour market flexibility. ^4^

### 4.2 Structural transformation and the agricultural trap

Agriculture’s dominance (45.9%) reflects an unfinished structural transformation. Workers exiting agriculture are predominantly absorbed into low-productivity informal services rather than formal manufacturing, constraining wage growth and perpetuating vulnerable employment. ^6, 27, 7, 3^ This phenomenon is most acute in East and West Africa, where very low *YUR* coexists with extensive subsistence employment and underemployment. Climate shocks compound these challenges, with rising temperatures reducing agricultural employment and accelerating labour reallocation towards manufacturing and services. ^37^ Addressing the agricultural trap requires strategies that modernise the sector while simultaneously creating alternatives in manufacturing and services for displaced agricultural workers. ^29, 30^

### 4.3 The educated unemployment paradox

The finding that upper secondary and tertiary graduates constitute 52.7% of unemployment constitutes compelling evidence of the educated unemployment paradox. ^8, 31^ This reflects poorly aligned curricula, limited higher-education–employer linkages, and private-sector absorptive constraints. The disproportionate impact on credentialled women underscores the role of discriminatory labour market institutions.

### 4.4 Gender and labour market inequality

The gender gap in *YUR* and *LFPR* is consistent with recent evidence on structural inequalities in African labour markets. ^11^ Women’s under-representation in indus-try limits access to productivity-enhancing formal employment. Expanding women’s access to industrial and technology-intensive occupations is essential and requires both supply-side (education, skills) and demand-side (anti-discrimination, quotas) interventions. ^1^

### 4.5 Informal economy and entrepreneurship

The prevalence of low *YUR* alongside high *NEET* rates in many sub-Saharan African countries reflects widespread necessity-driven informal employment rather than gen-uine labour market inclusion. Young people engage in informal activities including artisanal small-scale mining, street trading, and agricultural subsistence as survival strategies in the absence of formal opportunities. ^38, 39^ Entrepreneurship programs show promise but require comprehensive support including financial literacy, mentorship, and access to credit. ^40, 41, 42^ Public-private partnerships, such as the Youth Employment Service in South Africa, represent innovative models but require stronger post-placement support mechanisms. ^43, 44^

### 4.6 Limitations

Several limitations must be acknowledged. First, *ILO* modelled estimates carry inherent uncertainty for countries with outdated survey coverage; formal confidence intervals are unavailable, precluding strict inferential statistical testing. Second, the dataset does not capture employment quality (formal vs. informal, wage vs. self-employment), which is critical for comprehensive policy diagnosis. Third, the cross-sectional design precludes temporal trend analysis. Fourth, the 15–35 age bracket, whilst consistent with African Union definitions, is broader than the conventional 15–24 *ILO* window, affecting comparability. Future research should employ longitudinal panel designs, harmonised household survey microdata, multi-dimensional employment quality indices, and structural equation modelling. ^3^

### 4.7 Policy Implications

Evidence from this study supports the following priority interventions ^2, 1, 31, 36, 42, 43, 41^:

1. Accelerate structural economic transformation through industrial policy promoting labour-intensive manufacturing.
2. Expand quality TVET aligned with private-sector demand to address credential–employment mismatch, incorporating both technical and soft skills development. ^33, 34, 45^
3. Establish social protection floors for *NEET* youth to prevent human-capital atrophy. ^15^
4. Mainstream gender-transformative employment policies, including anti-discrimination legislation and childcare infrastructure. ^12^
5. Strengthen sub-regional labour market integration to enlarge effective labour markets, and leverage ICT and digital platforms to improve labour market matching. ^46^
6. Address energy infrastructure gaps as a complementary strategy, given evidence linking energy poverty to youth unemployment. ^47^

## Conclusions

This study provides the most comprehensive cross-national analysis of African youth labour markets to date, covering all 53 African Union member states in 2026 with harmonised *ILO* modelled estimates. The *YUR* of 6.91%, *NEET* rate of 19.23%, Gini coefficient of 0.61, agricultural employment dominance at 45.9%, a systematic gender gap, and the educated unemployment paradox collectively define a multi-dimensional structural challenge. All four hypotheses were confirmed. These findings underscore the imperative of moving beyond *YUR* as the primary metric towards multi-dimensional frameworks incorporating *NEET* rates, employment quality, sectoral composition, and gender. Evidence-based, structurally transformative, and gender-sensitive policies are indispensable for harnessing Africa’s demographic dividend.

## Data Availability

The data underlying the results of this study were retrieved from the Africa Youth Employment Clock (World Data Lab), an open-access platform available at https://africayouthjobs.io, which draws on ILO harmonised modelled labour market estimates (ILOSTAT). The four underlying datasets (employed_2026.xlsx, unemployed_2026.xlsx, inactive_2026.xlsx, student_2026.xlsx) have been deposited on Zenodo: World Data Lab. Youth Labour Market Dataset Africa, 2026. https://doi.org/10.5281/zenodo.19322273 Data are available under the terms of the Creative Commons Attribution 4.0 International license (CC-BY 4.0).
