# Youth Labour Market Participation in Africa: A Cross-National Analysis of Employment, Unemployment, Sectoral Distribution, and Educational Attainment Among the 15–35 Age Cohort, 2026

**DOI:** 10.12688/f1000research.179407.2

**Published:** 2026-08-03

**Authors:** Said Maizzou, Abdelhak Anajar, Mohamed Rahmo

**Affiliations:** 1 Laboratory of Strategy and Organization Management (LASMO), Hassan First University, BP 577, Settat, 26000, Morocco; 2 Independent Researcher, Casablanca, Morocco

**Keywords:** youth unemployment; NEET; Africa; labour force partic-ipation; sectoral employment; educational attainment; gender gap; ILO modelled estimates

## Abstract

**Background:**

Africa hosts the world’s largest youth cohort, yet its labour markets remain characterised by structural imbalances, agricultural dependence, gender inequalities, and educational credential mismatches. This study provides a cross-national analysis of youth labour market participation across the 54 African Union member states in 2026, disaggregated by sex, age group, economic sector, and educational attainment.

**Methods:**

A cross-sectional descriptive-correlational study was conducted using harmonised International Labour Organization (ILO) modelled estimates for youth aged 15–35 years. Youth unemployment (YUR), labour force participation (LFPR), and not-in-employment, education, or training (NEET) rates were analysed by country, sub-region, sex, age group, and education level. Pearson’s correlation assessed the association between YUR and NEET rate, while four nested ordinary least squares (OLS) models identified cross-country correlates of YUR.

**Results:**

Among 545.1 million African youth, 57.1% were employed, 4.2% unemployed, 15.0% inactive, and 23.6% in education. The continental YUR was 6.91%, ranging from 0.4% in Niger to 37.4% in Eswatini, with higher unemployment in North Africa (11.09%) than sub-Saharan Africa (6.40%). The NEET rate reached 19.23%, agriculture accounted for 45.9% of youth employment, and female unemployment exceeded male unemployment (7.56% vs. 6.40%). Upper secondary and tertiary graduates represented 52.7% of unemployed youth. YUR was strongly correlated with the NEET rate (r = 0.720, *p* < 0.001). OLS models showed that agricultural employment share was the strongest negative correlate of YUR (β = −0.255, *p* < 0.001), whereas the share of educated unemployed was positively associated with YUR ( *p* = 0.043).

**Conclusions:**

African youth labour markets exhibit substantial cross-country heterogeneity, persistent informality, and education–employment mismatches. Policies promoting structural transformation, skills alignment, gender equality, and social protection are needed. These findings highlight the limitations of unemployment rates alone as indicators of youth labour market performance.

## Introduction

### Background and rationale

Africa is undergoing an unprecedented demographic transition. By 2026, the continent hosts approximately 1.5 billion people, of whom an estimated 545 million are young persons aged 15–35 the largest youth cohort in the world. This demographic trajectory presents a dual opportunity: if adequately absorbed into productive employment, Africa’s youth could generate a demographic dividend capable of sustaining economic growth for decades; conversely, failure to integrate young people into the labour market risks perpetuating cycles of poverty, social exclusion, and political instability. ^1^ ^–^ ^5^

Despite economic growth averaging 4–5% per annum across sub-Saharan Africa since 2000, employment generation has consistently lagged behind labour-force expansion. A structural disconnect persists between the sectors absorbing the largest share of workers predominantly subsistence agriculture and the informal economy and the productive, high-value activities needed to deliver sustainable income gains. ^6^ ^,^ ^7^ Simultaneous rapid expansion of tertiary enrolment without commensurate growth in high-skilled employment has generated widespread credential–employment mismatch. ^8^ ^–^ ^11^

Gender represents a critical axis of labour market disadvantage. Young women face lower participation rates, higher unemployment, and greater concentration in precarious informal work relative to male counterparts, reflecting entrenched social norms, discriminatory hiring, and disproportionate care burdens. ^12^ ^–^ ^14^ The *NEET* indicator has emerged as a more comprehensive proxy for youth disengagement than unemployment alone, capturing both active job-seekers and discouraged workers who have with-drawn from the labour market entirely. ^15^ ^–^ ^18^

### Research questions

This study is guided by four research questions ( **RQ**):

**RQ**_1_ What is the distribution of labour market status employed, unemployed, inactive, in education/training among African youth aged 15–35 across all 53 countries in 2026?

**RQ**_2_ To what extent do *YUR*, *NEET* rates, and *LFPR* vary by country, sub-region, age group, and sex?

**RQ**_3_ What is the relationship between educational attainment and youth unemployment, and is there evidence of credential–employment mismatch?

**RQ**_4_ How does the sectoral distribution of youth employment differ by sex, and what structural implications does this hold for gender equity?

### Theoretical framework and literature review

Youth labour market outcomes in Africa are shaped by the interplay of structural economic transformation, human capital formation, demographic dynamics, and institutional constraints. Classical structural transformation theory posits that economic development entails a progressive reallocation of labour from low-productivity agriculture to higher-productivity manufacturing and services. ^6^ In Africa, this process remains incomplete: the majority of young workers are absorbed into subsistence agriculture and low-productivity informal services rather than formal-sector manufacturing, perpetuating vulnerable employment. ^7^ ^,^ ^19^

Several strands of the labour market literature are directly relevant to this study. First, the Harris– Todaro model of segmented labour markets predicts that formal-sector wage premiums attract migrants and new entrants into urban job search, generating unemployment as an equilibrium outcome a mechanism consistent with the high measured YUR in North African and Southern African economies where formal sectors are large but constrained. ^20^ ^–^ ^22^

Second, signalling and screening theories of education predict that when educational credentials become widely accessible without a corresponding expansion of skilled employment, the informational content of degrees diminishes, leading to credential inflation and educated unemployment the “diploma disease” phenomenon. ^9^ ^,^ ^23^ ^–^ ^25^

Third, search-and-matching models emphasise that frictional and structural mismatches between worker skills and employer requirements generate equilibrium unemployment that persists even in growing economies. ^8^ ^,^ ^26^ Gender inequalities in African labour markets have been extensively documented. Women face lower participation rates, higher unemployment, and greater concentration in precarious informal work, reflecting entrenched social norms, discriminatory hiring practices, and disproportionate unpaid care burdens. ^12^ ^,^ ^13^ The NEET indicator has emerged as a more comprehensive proxy for youth disengagement than unemployment alone, capturing both active job-seekers and discouraged workers who have withdrawn from the labour market entirely. ^15^ ^–^ ^18^ Recent evidence indicates that motherhood is among the strongest predictors of NEETstatus across countries, amplifying gender disparities in youth labour market attachment. ^27^

The African youth demographic context adds further urgency. With the continent’s population projected to double by 2050, youth will constitute the largest cohort of both new labour-market entrants and potential emigrants, generating migratory pressures that interact with domestic labour market conditions. ^28^ The relationship between education and employment is particularly complex in this context: while higher education is widely perceived as a pathway to formal employment, rapid educational expansion has outpaced high-skilled job creation, generating the paradox of educated unemployment observed across the continent. ^11^ ^,^ ^29^ Income inequality has been identified as a key accelerator of youth unemployment across African countries, while political instability exerts additional upward pressure through its effects on investment and labour market flexibility. ^4^ ^,^ ^5^

### Hypotheses

Building on theory and recent empirical evidence, ^30^ ^–^ ^32^ this study formulates four testable propositions ( **H**) grounded in descriptive comparisons:

H _1_
*YUR* is significantly higher in North Africa than in sub-Saharan Africa.

H _2_
Female youth face systematically higher *YUR* and lower *LFPR* than male youth.

H _3_
Youth with upper secondary and tertiary qualifications account for a disproportionate share of unemployment relative to their population weight.

H _4_
*YUR* and NEET rate are positively correlated at country level.

### Significance and scope

Pan-African comparative analyses simultaneously examining *YUR*, *NEET*, *LFPR*, sectoral structure, and educational attainment with full sex disaggregation across all 53 African countries remain scarce. ^1^ ^,^ ^30^ This study fills that gap and provides an evidence base for policies aligned with the African Union’s Agenda 2063 and United Nations Sustainable Development Goal 8 (Decent Work and Economic Growth).

## Methods

### Study design

A cross-sectional, descriptive-correlational study design was employed. Analysis was conducted at country level across 54 African Union member states for the calendar year 2026. The study follows the STROBE (Strengthening the Reporting of Observational Studies in Epidemiology) guidelines, adapted for secondary aggregate data. ^33^ Geospatial analysis follows Bayesian approaches that account for spatial dependence and heterogeneity across countries and sub-regions. ^34^

### Data source

All data derive from *ILO* harmonised modelled labour market estimates (ILOSTAT, 2024 release), ^35^ accessed via the Africa Youth Employment Clock platform (World Data Lab). These estimates are produced through a multi-step framework combining national household survey microdata, administrative records, and econometric imputation for countries with incomplete or outdated survey coverage, ensuring cross-national comparability. ^36^

**Caveat on modelled estimates.** Because the most recent observed data in the 2024 ILOSTAT release span 2022–2023 for most countries, the 2026 values reported in this study are modelled projections rather than directly observed labour market outcomes. All estimates therefore carry prediction uncertainty that is not captured in the point estimates reported below. Readers should interpret the findings as indicative projections subject to revision when observed survey data become available.

The target population is youth aged 15–35 years, consistent with the African Union’s definition of youth. Four labour market status variables were examined:

• **Employed**: performed *≥*1 hour of paid or self-employed work in the reference period; disaggregated by country, single year of age, sex, and ISIC Rev.4 sector (aggregated as Agriculture, Industry, Services).
• **Unemployed**: not in employment, actively seeking work, and available to start; disaggregated by country, age, sex, and educational attainment (no education; primary; lower secondary; upper secondary; tertiary).
• **Inactive**: neither employed nor seeking work (including discouraged workers); disaggregated analogously.
• **Students**: currently enrolled in formal or non-formal education or training; disaggregated analogously.

### Outcome indicators

Three primary indicators were computed for each country:

$$\mathrm{YUR}=\frac{\text{Unemployed}}{\text{Employed}+\text{Unemployed}}\times 100$$ (1)

$$\text{LFPR}=\frac{\text{Employed}+\text{Unemployed}}{\text{Total Youth}}\times 100.$$ (2)

$$\text{NEET}=\frac{\text{Unemployed}+\text{Inactive}}{\text{Total Youth}}\times 100$$ (3)

where “Inactive” refers to economically inactive youth who are neither employed, unemployed, nor in education or training, consistent with the standard *ILO*NEET definition. Students enrolled in education or training are excluded from both the numerator and the “Inactive” category. Definitions conform to *ILO* resolutions adopted at the 13th and 19th International Conferences of Labour Statisticians. ^36^

Sub-regional aggregates were computed for North Africa (Algeria, Egypt, Libya, Morocco, Sudan, Tunisia) and sub-Saharan Africa (the remaining 48 countries).

### Statistical analysis

All analyses were performed in Python 3.12 using *pandas* v2.2, *NumPy* v1.26, *statsmodels* v0.14, and *scipy* v1.17; figures were produced with *matplotlib* v3.10 and *seaborn* v0.13 at a minimum resolution of 300 dpi to ensure legibility of all numerical labels and colour coding at print size. To test H _4_, Pearson’s correlation coefficient *r* was computed between country-level *YUR* and *NEET* rate ( *n* = 54; significance threshold *α* = 0.05). Cross-country inequality in *YUR* was quantified using the Gini coefficient.

To extend the analysis beyond bivariate associations, four nested ordinary least squares (OLS) regression models were estimated with *YUR* as the dependent variable. *NEET* rate and *LFPR* were excluded as predictors because *NEET* is a mathematical function of *YUR* and *LFPR* ( *NEET* = (1 *− LFPR*) + ( *LFPR× YUR*)); their inclusion would render the regression tautological. The models therefore use only conceptually independent predictors:

**M**_1_ Sectoral model: YUR = $\beta_{0}+\beta_{1}$ AgricultureShare + ε .

**M**_2_ Regional model: adds North Africa dummy to M _1_.

**M**_3_ Full model: adds gender gap in *YUR* and upper-secondary/tertiary share of unemployment to M _2_.

**M**_4_ Parsimonious model: retains only predictors significant at *α* = 0.05 in M _3_.

Model assumptions were assessed via variance inflation factor (VIF, threshold *<*5) and residual diagnostics. All descriptive statistics are reported to one decimal place. Statistical significance is denoted as ^∗^ *p* < 0.05, ^∗∗^ *p* < 0.01, ^∗∗∗^ *p* < 0.001. Continental averages and the Gini coefficient are unweighted (each country contributes equally), which should be considered when interpreting aggregate figures.

### Ethical considerations

This study uses exclusively publicly available, aggregate, anonymized *ILO* modelled estimates. No primary data collection involving human participants or animals was undertaken. Formal ethical approval was not required under applicable institutional guidelines. The dataset is freely accessible at https://africayouthjobs.io.

## Results

### Overall labour market status distribution (RQ _1_)

The total African youth population aged 15–35 comprised an estimated 545.1 million individuals across 53 countries in 2026 ( Figure 1). Of these, 311.5 million (57.1%) were employed, 23.1 million (4.2%) were unemployed, 81.7 million (15.0%) were economically inactive, and 128.8 million (23.6%) were in education or training. The continental *YUR* was 6.91% and the *LFPR* was 61.38%. The *NEET* rate reached 19.23%, representing approximately 104.8 million youth neither employed nor in education/training a population at elevated risk of long-term human-capital atrophy. ^15^ ^,^ ^16^

**Figure 1.**
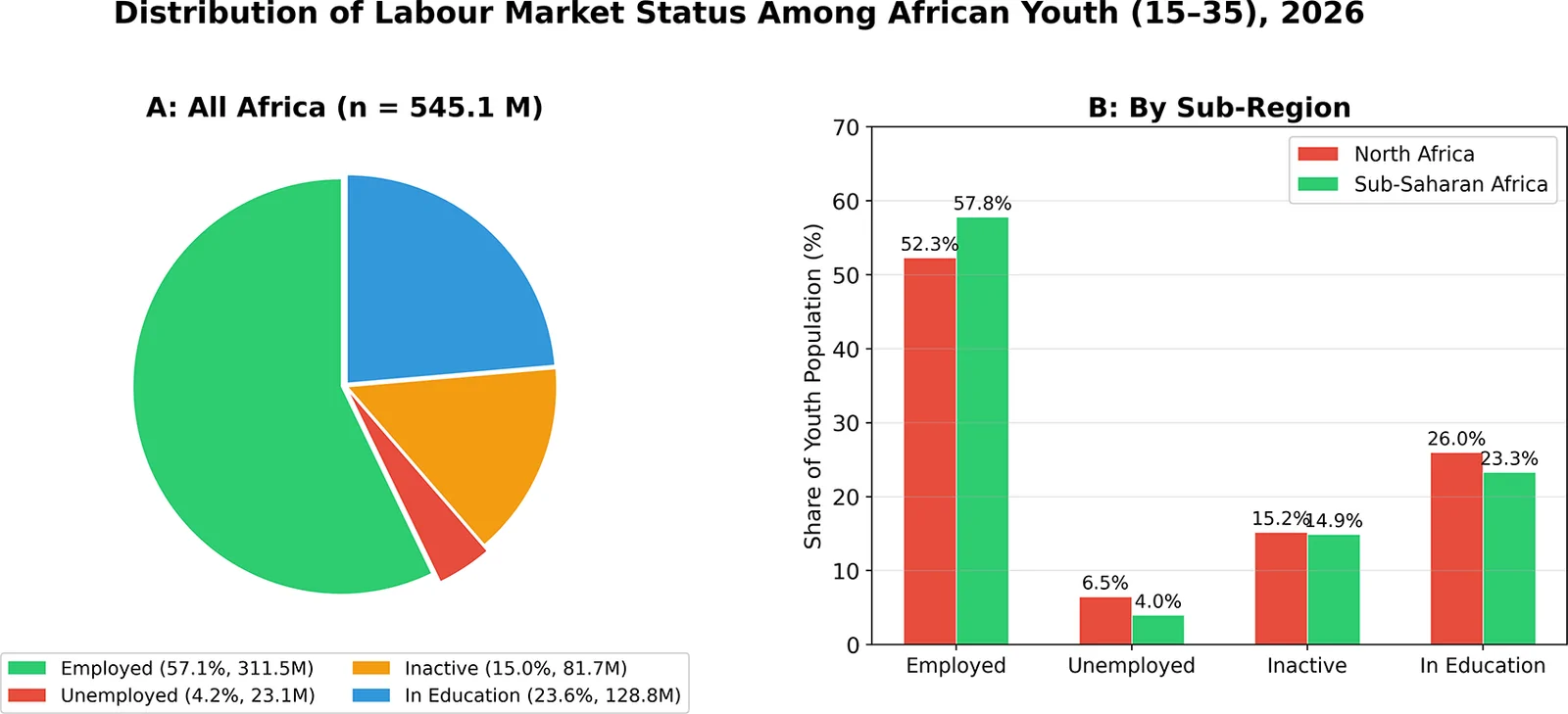
Distribution of labour market status among African youth (15–35 Years), 2026. Panel (A): proportional distribution across four statuses for all 53 countries combined (total = 545.1 million). Panel (B): sub-regional comparison between North Africa ( *n* = 6) and Sub-Saharan Africa ( *n* = 47). Source: Author’s calculations based on (World Data Lab 2026).

### Cross-country heterogeneity and sub-regional patterns (RQ _2_; H _1_)

Figure 2 illustrates the pronounced cross-country heterogeneity in *YUR.* Rates range from 37.4% in Eswatini to 0.4% in Niger a 93-fold differential. The Gini coefficient for *YUR* across 53 countries equals 0.61, indicating very high cross-country inequality. The 15 highest- *YUR* countries include Eswatini (37.4%), South Africa (36.5%), Djibouti (33.6%), Botswana (29.2%), and the Republic of the Congo (25.1%), sharing characteristics of structurally constrained formal sectors relative to growing youth labour supply. ^22^ ^,^ ^32^

**Figure 2.**
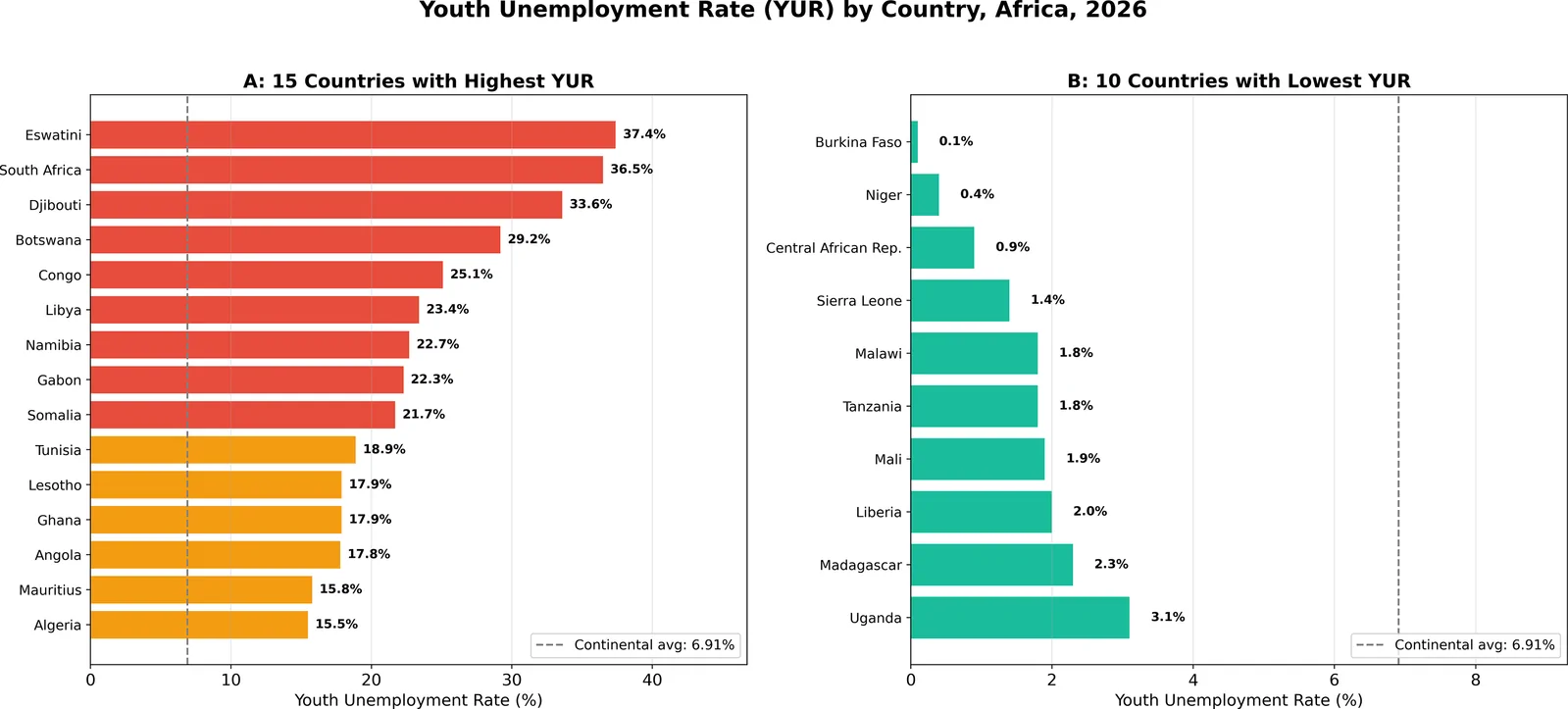
Youth unemployment rate (YUR) by country, Africa, 2026. Panel (A): 15 countries with highest *YUR.* Panel (B): 10 countries with lowest *YUR.* Dashed vertical line = continental average (6.91%). Colour coding: red *≥*20% (critical); amber 10–19% (high); teal *<*10% (moderate/low). Source: Author’s calculations based on (World Data Lab 2026).

Consistent with **H**_1_, North Africa recorded a substantially higher *YUR*(11.09%) than sub-Saharan Africa (6.40%). This descriptive comparison is consistent with H _1_, although the small number of North African countries ( *n* = 6) limits the statistical power of formal hypothesis tests. These patterns reflect the distinctive characteristics of North African labour markets: large but saturated public sectors, limited private-sector dynamism, and elevated rates of educated unemployment, especially among women. ^21^ ^,^ ^31^ Paradoxically, several sub-Saharan African countries record very low *YUR* (Niger 0.4%, Burundi 1.1%) yet high *NEET* rates (13.9% and 9.5%, respectively), reflecting necessity employment where the absence of social protection compels any form of work regardless of productivity. ^2^ ^,^ ^37^ Table 1 presents the full set of indicators for all 54 countries (selected, ranked by *YUR*).

**Table 1.** Youth labour market indicators by country, Africa, 2026 (selected countries, Ranked by YUR).

Country	YUR (%)	NEET (%)	LFPR (%)	Employed (000 s)	Unemployed (000 s)
Eswatini	37.4	37.3	46.9	139	83
South Africa	36.5	33.6	53.2	7 727	4 434
Djibouti	33.6	40.9	28.1	81	41
Botswana	29.2	31.3	63.2	444	183
Rep. of Congo	25.1	24.9	60.7	1 029	345
Libya	23.4	25.4	41.2	764	233
Namibia	22.7	25.2	51.9	400	117
Gabon	22.3	24.4	43.3	298	85
Somalia	21.7	36.9	28.9	1 505	417
Tunisia	18.9	18.5	44.9	1 329	311
Lesotho	17.9	31.4	53.0	387	84
Angola	17.8	21.0	69.1	7 731	1 671
Algeria	15.5	35.0	40.0	4 696	859
South Sudan	14.7	23.1	69.7	2 347	405
Morocco	11.6	27.0	42.4	4 661	612
Rwanda	12.7	21.9	62.0	2 988	435
Egypt	8.9	20.6	41.0	14 775	1 451
Mozambique	7.9	16.3	75.1	9 047	781
Kenya	7.3	16.6	57.7	11 285	895
Ethiopia	3.8	14.5	66.0	32 179	1 268
Nigeria	3.8	11.1	77.3	62 139	2 463
Uganda	3.1	11.3	78.2	15 192	491
Tanzania	1.8	9.3	80.3	19 996	369
Niger	0.4	13.9	77.4	7 630	33
**Continental total**	**6.9**	**19.2**	**61.4**	**311 499**	**23 114**

Notes: YUR = Youth Unemployment Rate; NEET = Not in Employment, Education or Training; LFPR = Labour Force Participation Rate. Source: Author’s calculations based on (World Data Lab 2026).

### Age-group differentials (RQ _2_)

Youth unemployment exhibits a non-linear age profile. Rates were highest in the 20–24 cohort (9.52%) and in the 15–19 cohort (9.41%), declining to 4.83% for ages 25–29 and 5.19% for ages 30–35. This pattern is broadly consistent with transitional unemployment among recent school-leavers. ^1^ ^,^ ^8^ However, the interpretation of these age-group differentials must account for the composition of the youth population. In particular, the unemployment rate may not be an informative indicator of labour market outcomes during the transition from education to work, because students can be simultaneously enrolled in education and actively seeking employment; their responses to survey questions on labour force participation are difficult to predict and may inflate measured unemployment in younger cohorts. ^38^ The relatively high YUR observed in the 15–19 group (9.41%) should therefore be interpreted with caution, as it partly reflects high labour force attachment among students rather than purely involuntary joblessness. The modest uptick for the 30–35 group is consistent with labour-market scarring, whereby prolonged early unemployment reduces long-run employability. ^15^ Evidence from hysteresis tests confirms that youth unemployment persistence varies significantly across African countries and income levels. ^39^

### 3.4. Sectoral distribution and gender patterns (RQ2, RQ4; H2)

Agriculture dominated youth employment at 45.9% (143.1 million), followed by services at 40.0% (124.6 million) and industry at 14.1% (43.8 million) ( Table 2). Agricultural dominance is most pronounced in Niger, Tanzania, and Ethiopia, where low measured *YUR* coexists with extensive subsistence farming. Countries with more diversified structures South Africa, North African states record higher industrial and services shares alongside higher formal unemployment. ^6^ ^,^ ^7^ ^,^ ^19^ The agriculture sector represents both a repository of last-resort employment and a potential engine for inclusive development if adequately modernised and linked to youth-inclusive value chains. ^40^ ^–^ ^42^

**Table 2.** Youth employment by economic sector and sex, Africa, 2026.

Sector	Female (000 s)	Male (000 s)	Total (000 s)	Share of total (%)
Agriculture	62 697	80 423	143 120	45.9
Services	59 789	64 806	124 595	40.0
Industry	13 770	30 014	43 784	14.1
**Total**	**136 256**	**175 243**	**311 499**	**100.0**

Source: Author’s calculations based on (World Data Lab 2026).

Supporting **H**_2_, female *YUR*(7.56%) exceeded male *YUR*(6.40%), a gap of 1.16 percentage points at the continental level. This descriptive gap is consistent with H _2_, although individual country-level patterns may vary. Gender disparities are further visible in the *NEET* rate, where female youth recorded substantially higher disengagement than male youth, reflecting the combined effects of lower participation, higher unemployment, and greater exclusion from education and training among young women. Motherhood has been identified as one of the strongest predictors of *NEET* status in cross-country analyses, suggesting that fertility-related barriers are a critical driver of gender disparities in youth labour market attachment. ^27^

Gender disparities are also evident in the sectoral distribution ( Figure 3; Table 2). Women were markedly under-represented in industry (10.1% of female employment vs. 17.1% of male employment), whilst services claimed a higher share among women (43.9%) than men (37.0%), reflecting the concentration of women in informal service activities, domestic work, and petty trade. ^12^ ^,^ ^13^ Female employment totaled 136.3 million versus 175.2 million for males.

**Figure 3.**
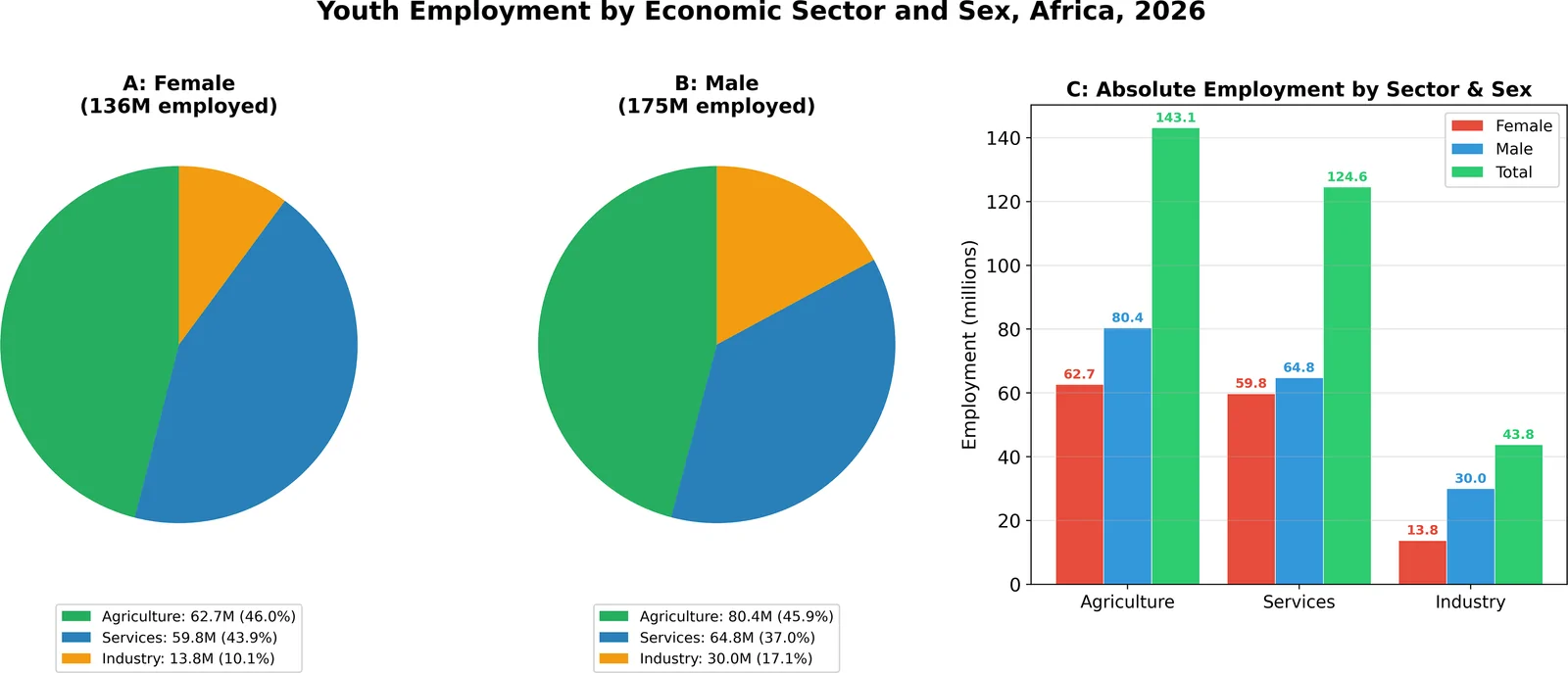
Youth employment by economic sector and sex, Africa, 2026. Panels (A)–(B): proportional sectoral distribution for female and male employed youth. Panel (C): absolute figures by sector and sex (millions). Sectors: Agriculture (green), Industry (amber), Services (navy). Source: Author’s calculations based on (World Data Lab 2026).

### Unemployment by educational attainment (RQ _3_; H _3_)

Figure 4 and Table 3 are consistent with **H**_3_. Upper secondary graduates constituted 32.9% of all unemployed youth (7.6 million) and tertiary graduates 19.8% (4.6 million); together, 52.7% of total unemployment. However, a rigorous test of credential– employment mismatch requires comparing these shares to the educational distribution of the labour force (rather than of the unemployed alone); this comparison is not possible with the aggregate data available. Furthermore, this pattern may be partly driven by higher labour force participation rates among more educated youth: because the unemployment rate is conditional on activity, higher search intensity and stronger formal-sector attachment among educated youth mechanically expand the pool of individuals who can be counted as unemployed. ^38^

**Figure 4.**
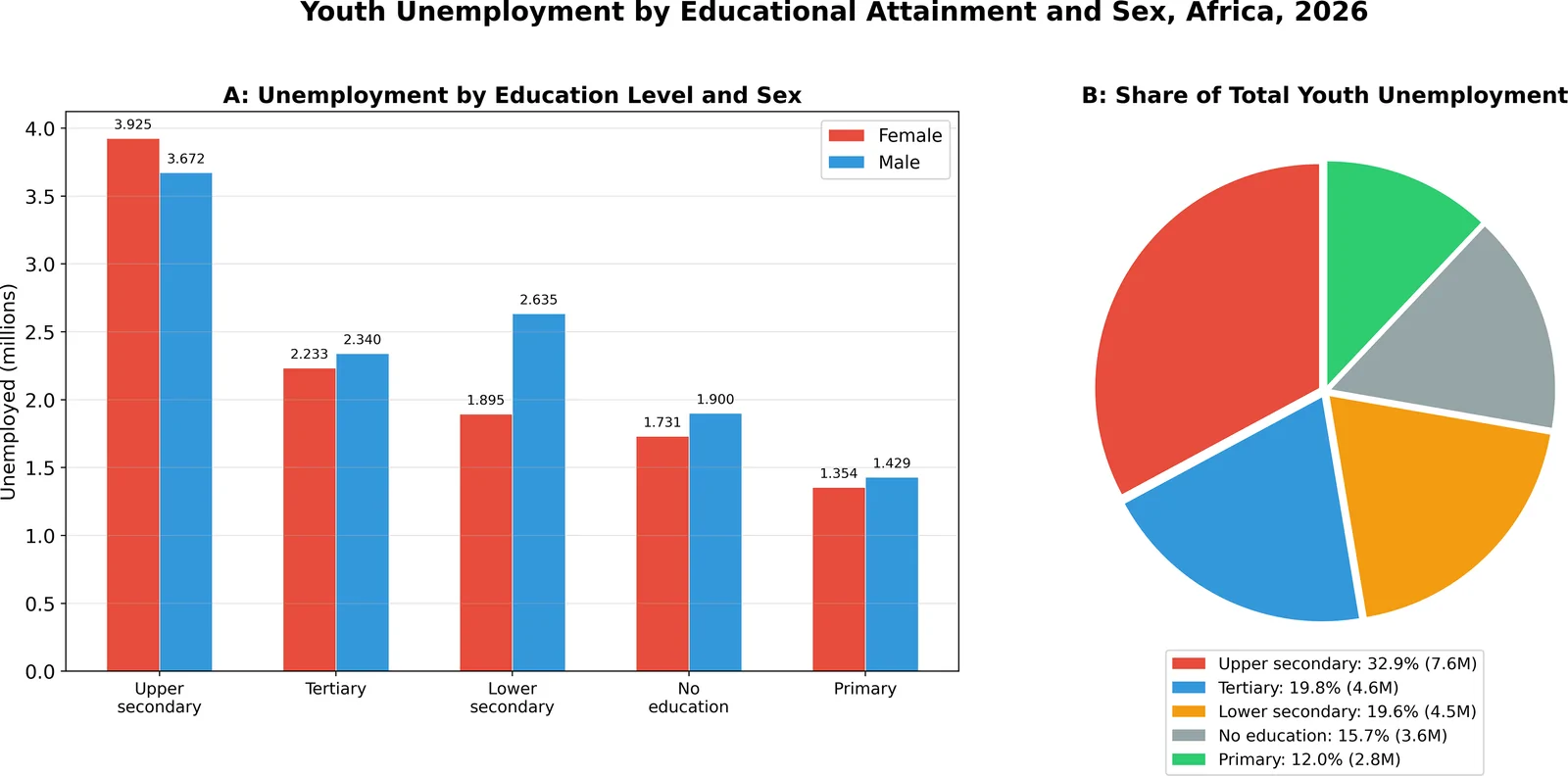
Youth unemployment by educational attainment and sex, Africa, 2026. Panels (A)–(B): proportional sectoral distribution for female and male employed youth. Panel (C): absolute figures by sector and sex (millions). Sectors: Agriculture (green), Industry (amber), Services (navy). Source: Author’s calculations based on (World Data Lab 2026).

**Table 3.** Youth unemployment by educational attainment and sex, Africa, 2026.

Education Level	Female (000 s)	Male (000 s)	Total (000 s)	Share of total (%)
Upper secondary	3 925	3 672	7 597	32.9
Tertiary	2 233	2 340	4 573	19.8
Lower secondary	1 895	2 635	4 530	19.6
No education	1 731	1 900	3 631	15.7
Primary	1 354	1 429	2 783	12.0
**Total**	**11 138**	**11 976**	**23 114**	**100.0**

Source: Author’s calculations based on (World Data Lab 2026).

By contrast, youth without formal education accounted for only 15.7% (3.6 million). This *educated unemployment paradox* reflects a combination of structural mismatches between curricula and private-sector demand and differential activity patterns across educational levels. ^8^ ^–^ ^10^ ^,^ ^29^ ^,^ ^43^ The persistent skills gap particularly in digital and soft skills constrains even educated youth from transitioning into productive employment. ^44^ ^–^ ^46^

By sex, women with upper secondary credentials constituted 35.3% of female unemployment (3.9 million) versus 30.7% for males (3.7 million), reinforcing the compounding role of gender discrimination in amplifying educational mismatch. ^12^ ^,^ ^13^

### YUR–NEET correlation (H _4_)

Pearson’s correlation between country-level *YUR* and *NEET* rate ( *n* = 54) yielded *r* = 0.720 ( *p* = *<* 0.001); Spearman’s rank correlation yielded *ρ* = 0.694 ( *p <* 0.001), confirming that the association is robust to distributional assumptions ( Figure 5). A positive association between formal labour-market stress and broader youth disengagement is therefore evident. Notable deviations from the trend include Algeria ( *YUR* 15.5%, *NEET* 35.0%) high on both dimensions and Chad ( *YUR* 1.2%, *NEET* 31.3%) low unemployment yet extreme inactivity reflecting necessity employment in the absence of social protection.

**Figure 5.**
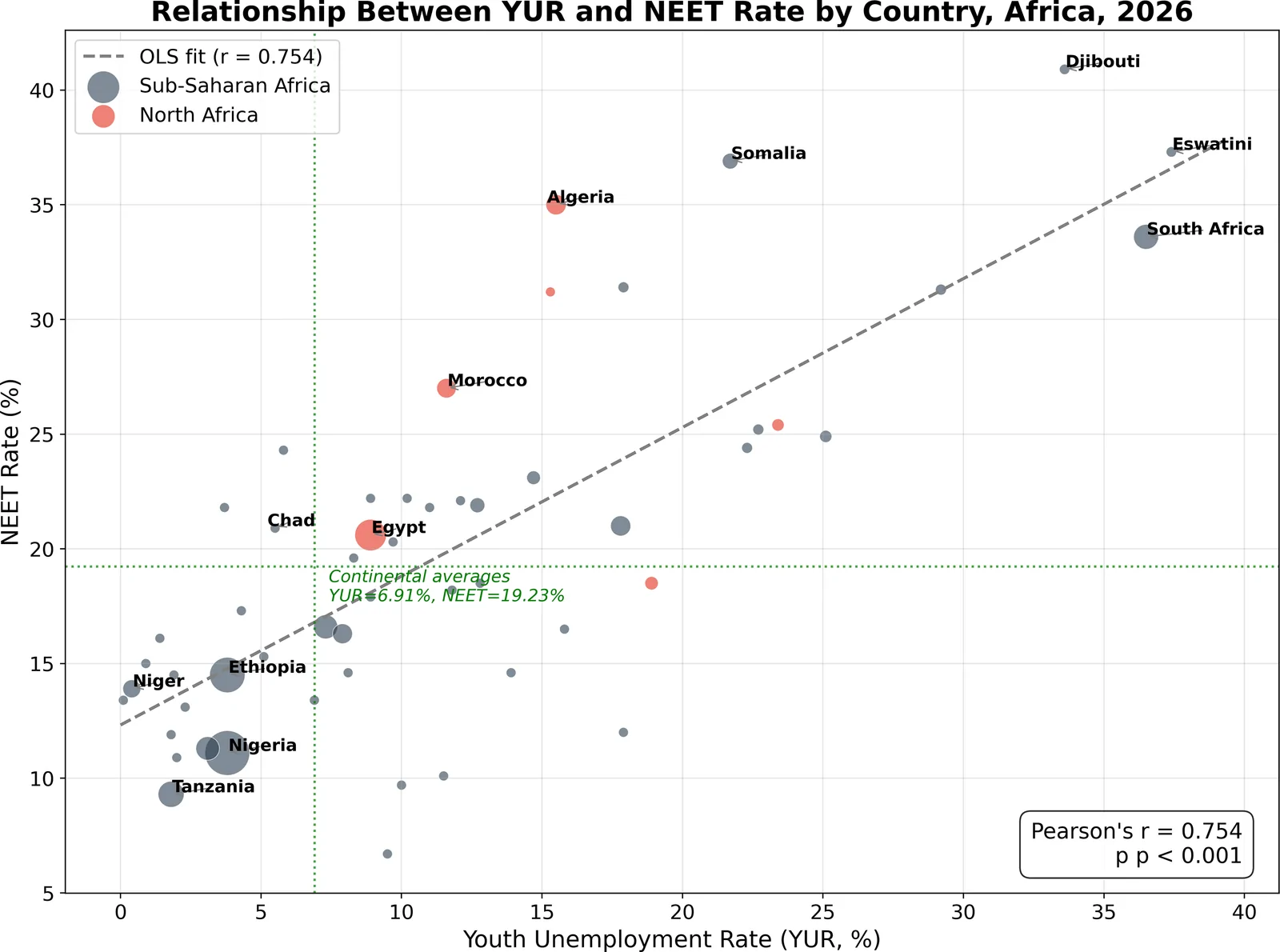
Relationship between youth unemployment rate (YUR) and NEET rate by country, Africa, 2026. Bubble size is proportional to youth population aged 15–35. Red = North Africa; navy = Sub-Saharan Africa. Dotted lines denote continental averages on each axis. Dashed regression line: *r* = 0.720, *p* = *<* 0.001 ( *n* = 53). Source: Author’s calculations based on (World Data Lab 2026).

### Regression analysis: Correlates of YUR

Table 4 presents the results of four nested OLS models examining cross-country correlates of *YUR.* Because *NEET* rate and *LFPR* are mechanically related to *YUR* (see Discussion), these variables were excluded as predictors; only conceptually independent regressors are included.

**Table 4.** Ordinary Least Squares Regression Results: Correlates of Youth Unemployment Rate (YUR) Across African Countries, 2026.

Predictor	Model 1 (Sector)	Model 2 (+Region)	Model 3 (Full)	Model 4 (Parsimonious)
Intercept	22.9954*** (2.4398)	25.0956*** (2.9157)	10.3656 (7.0888)	12.3482* (6.0971)
Agriculture share of employment (%)	−0.2550*** (0.0502)	−0.2905*** (0.0569)	−0.2986*** (0.0560)	−0.2687*** (0.0495)
North Africa (dummy)	—	−4.8132 (3.7136)	−10.2980 (5.8161)	—
Gender gap in YUR (Female − Male, percentage points)	—	—	1.7827 (1.4929)	—
Upper secondary/tertiary share of unemployed (%)	—	—	0.2469* (0.1189)	0.2259 (0.1191)
R ^2^	0.331	0.353	0.414	0.375
Adjusted R ^2^	0.319	0.327	0.366	0.351
**Observations (N)**	**54**	**54**	**54**	**54**

Notes: Standard errors in parentheses. Dependent variable: YUR (%). Significance: ∗∗∗p < 0.001; ∗∗p < 0.01; ∗p < 0.05. All models estimated via OLS (n = 54 African Union member states with ILO data). Model 1 includes agriculture share only; Model 2 adds the North Africa regional dummy; Model 3 is the full specification; Model 4 retains only predictors significant at p < 0.05 in M3. NEET rate and LFPR are excluded because they are mechanically related to YUR through the identity NEET = (1 − LFPR) + (LFPR × YUR).

In the sectoral model (M _1_), agriculture employment share alone explained 33.1% of cross-country *YUR* variance (adjusted *R* ^2^ = 0.319). Agriculture share exerted a strong negative effect ( *β* = *−*0.255, *p <* 0.001), confirming that countries with larger agricultural sectors exhibit systematically lower measured *YUR* consistent with the necessity-employment hypothesis ( **H**_2_).

Adding the North Africa dummy in M _2_ did not significantly improve model fit (adjusted *R* ^2^ = 0.327; *∆R* ^2^ = 0.008; North Africa *β* = *−*4.813, *p* = 0.201). This indicates that once sectoral structure is accounted for, North African countries do not significantly differ from sub-Saharan countries in *YUR.* This result qualifies **H**_1_: the higher raw *YUR* in North Africa (11.09% vs. 6.40%) is attributable to differences in sectoral composition rather than region-specific labour market institutions per se. A similar pattern emerges when comparing the North Africa dummy across models: its coefficient becomes non-significant ( *p* = 0.083) once agricultural share is controlled for, suggesting that the apparent regional effect is mediated by sectoral structure.

The full model (M _3_; adjusted *R* ^2^ = 0.366) retained agriculture share as the dominant predictor ( *β* = *−*0.299, *p <* 0.001).

The share of unemployed with upper-secondary or tertiary education was positively and significantly associated with *YUR* ( *β* = 0.247, *p* = 0.043), providing partial multivariate support for the credential—employment mismatch ( **H**_3_). The gender gap in *YUR* was not significant ( *p* = 0.238), and the North Africa dummy remained non-significant ( *p* = 0.083). The log-transformed specification (M _5_) confirmed the robustness of these findings: agriculture share ( *β* = *−*0.030, *p <* 0.001) and upper-secondary education share ( *β* = 0.028, *p* = 0.006) were both significant predictors of log- *YUR*, while gender gap ( *p* = 0.181) and North Africa ( *p* = 0.080) remained non-significant.

The parsimonious model (M _4_) retained agriculture share ( *p <* 0.001) and the education variable (marginally significant, *p* = 0.064; adjusted *R* ^2^ = 0.351). All VIF values were below 3.4, indicating no multicollinearity concerns. Residual diagnostics indicated non-normality in the untransformed models (Jarque–Bera *p <* 0.001 for M _1_–M _4_), but the log transformed specification (M _5_) satisfied the normality assumption (Jarque–Bera *p* = 0.648). Together, these results demonstrate that agricultural employment share is the most robust correlate of cross-country *YUR* variation, while educational composition of unemployment shows a consistent positive association in better-specified models.

## Discussion

### Principal findings in context

This study provides a harmonised, pan-African portrait of youth labour markets in 2026, with findings broadly consistent with all four propositions. The continental *YUR* of 6.91% (Gini coefficient 0.61) masks a *NEET* rate of 19.23%, a 93-fold country-level *YUR* range, a strong YUR–NEET correlation ( *r* = 0.720), and persistent structural imbalances. ^2^ ^,^ ^47^ These findings are consistent with structural transformation theory, which predicts that economies with large agricultural sectors generate low measured unemployment because the absence of social protection compels necessity-driven employment in low-productivity activities even when productive, remunerative work is unavailable. ^6^ ^,^ ^7^ ^,^ ^37^ The regression analysis confirms this mechanism: agriculture share alone explains 33.1% of cross-country *YUR* variance ( *β* = *−*0.255, *p <* 0.001), and the negative coefficient is robust across all specifications. This finding is consistent with the Harris–Todaro framework, wherein segmented labour markets produce divergent unemployment outcomes depending on the relative sizes of formal and informal sectors. ^20^

The share of unemployed with upper-secondary or tertiary education was positively associated with *YUR* in the full model ( *p* = 0.043), providing partial multivariate evidence for credential employment mismatch ( **H**_3_). This pattern aligns with signalling and screening theories of education, which predict that credential inflation occurs when educational expansion outpaces the creation of high-skilled employment. ^23^ ^,^ ^24^ Notably, the North Africa sub-Saharan gap in *YUR* became non-significant once agriculture share was controlled for ( *p* = 0.201), indicating that the higher raw *YUR* in North Africa is attributable to sectoral composition rather than region-specific institutions per se. These findings underscore the inadequacy of *YUR* alone as a policy metric and highlight the need for multi-dimensional frameworks. ^15^ ^–^ ^17^

### Structural transformation and the agricultural trap

Agriculture’s dominance (45.9%) reflects an unfinished structural transformation. Workers exiting agriculture are predominantly absorbed into low-productivity informal services rather than formal manufacturing, constraining wage growth and perpetuating vulnerable employment. ^3^ ^,^ ^6^ ^,^ ^7^ ^,^ ^19^ This phenomenon is most acute in East and West Africa, where very low *YUR* coexists with extensive subsistence employment and underemployment. Climate shocks compound these challenges, with rising temperatures reducing agricultural employment and accelerating labour reallocation towards manufacturing and services. ^48^ Addressing the agricultural trap requires strategies that modernise the sector while simultaneously creating alternatives in manufacturing and services for displaced agricultural workers. ^41^ ^,^ ^42^

### The educated unemployment paradox

The finding that upper secondary and tertiary graduates constitute 52.7% of unemployment constitutes compelling evidence of the educated unemployment paradox. ^8^ ^,^ ^9^ ^,^ ^29^ This pattern whereby higher educational attainment is associated with higher measured unemployment is characteristic of transition economies and reflects the interaction of several mechanisms. First, signalling and screening theories predict that when educational credentials become widely accessible without a corresponding expansion of skilled employment, the informational content of degrees diminishes, leading to credential inflation and educated unemployment the “diploma disease” phenomenon. ^23^ ^–^ ^25^ Second, higher educational attainment is typically associated with higher labour force participation rates, which mechanically increases the pool of individuals who can be counted as unemployed. ^38^ The observed pattern may therefore reflect, at least in part, higher search intensity and stronger formal-sector attachment among educated youth rather than or in addition to genuine skills mismatch. Third, the rapid expansion of tertiary enrolment in Africa has outpaced the growth of high-skilled employment, creating a structural surplus of credentialed workers. ^11^ A definitive test of the credential–employment mismatch hypothesis would require comparing the educational composition of the unemployed with that of the employed labour force; the aggregate data available do not permit this comparison. Nevertheless, the persistent skills gap in digital and soft skills constrains even educated youth from transitioning into productive employment, and the disproportionate impact on credentialled women underscores the compounding role of gender-based labour market discrimination. ^12^ ^,^ ^13^ ^,^ ^44^ ^–^ ^46^

### Gender and labour market inequality

The gender gap in *YUR* (7.56% female vs. 6.40% male) is consistent with recent evidence on structural inequalities in African labour markets. ^12^ ^,^ ^13^ However, attributing this gap solely to discrimination is insufficient. The labour market’s capacity to absorb workers varies according to the characteristics of the workforce, not only gender per se. Women’s concentration in services rather than industry partly reflects differential sectoral composition of female and male labour supply, as well as occupational segregation driven by social norms and care responsibilities. Recent cross-country evidence identifies motherhood as one of the strongest predictors of *NEET* status among young women, ^27^ suggesting that fertility transitions and childcare infrastructure are critical mediating factors. Women’s under-representation in industry (10.1% vs. 17.1% for males) limits access to productivity-enhancing formal employment, while the higher share of women in services (43.9% vs. 37.0%) reflects concentration in informal activities, domestic work, and petty trade. ^12^ ^–^ ^14^ Expanding women’s access to industrial and technology-intensive occupations requires both supply-side (education, skills) and demand-side (anti-discrimination legislation, childcare infrastructure) interventions. ^1^

### Informal economy, necessity employment, and migration

The prevalence of low *YUR* alongside high *NEET* rates in many sub-Saharan African countries reflects widespread necessity-driven informal employment rather than genuine labour market inclusion. Young people engage in informal activities including artisanal small-scale mining, street trading, and agricultural subsistence as survival strategies in the absence of formal opportunities. ^49^ ^,^ ^50^ This pattern is consistent with the interpretation that low measured unemployment in agriculture-dominated economies reflects the absence of social protection rather than genuine labour market strength. ^2^ ^,^ ^37^

Africa’s rapid population growth will further intensify migratory pressures. Young people constitute the largest cohort of potential emigrants from the continent, and both internal rural–urban migration and international migration interact with domestic labour market conditions in complex ways. The Harris–Todaro model predicts that migration decisions are driven by expected income differentials between sectors and regions, generating urban unemployment as an equilibrium outcome. ^20^ In countries where formal sectors are saturated and informal employment is the norm, emigration may serve as a safety valve—but also as a brain-drain mechanism that depletes human capital. ^28^ The current analysis does not capture migration flows, representing an important limitation for future research.

Entrepreneurship programs show promise but require comprehensive support including financial literacy, mentorship, and access to credit. ^51^ ^–^ ^53^ Public-private partnerships, such as the Youth Employment Service in South Africa, represent innovative models but require stronger post-placement support mechanisms. ^54^ ^,^ ^55^

### Limitations

Several limitations must be acknowledged. First, *ILO* modelled estimates carry inherent uncertainty for countries with outdated survey coverage; formal confidence intervals are unavailable, precluding strict inferential statistical testing. Second, the dataset does not capture employment quality (formal vs. informal, wage vs. self-employment), which is critical for comprehensive policy diagnosis. Third, the cross-sectional design precludes temporal trend analysis and limits causal inference; the regression estimates identify associations, not causal effects, and may be biased by omitted variables (e.g., institutional quality, labour market regulations, or macroeconomic conditions) for which country-level proxies are not available in the *ILO* dataset. Fourth, the 15–35 age bracket, whilst consistent with African Union definitions, is broader than the conventional 15–24 *ILO* window, affecting comparability. Fifth, the aggregate data do not allow a rigorous test of credential employment mismatch, which requires comparing the educational distribution of the unemployed with that of the employed labour force. Sixth, the unemployment rate may not be an informative indicator of labour market outcomes for the 15–19 age group, where many individuals are simultaneously studying and searching for work; their survey responses regarding labour force participation are difficult to interpret. ^38^ Seventh, the analysis does not capture migration flows, which may significantly affect youth labour market dynamics in high-emigration countries.

Future research should employ longitudinal panel designs, harmonised household survey microdata, multi-dimensional employment quality indices, instrumental variable approaches, and structural equation modelling. ^3^ ^,^ ^8^

### Policy implications

Evidence from this study, grounded in the preceding analysis and discussion, supports the following priority interventions ^1^ ^,^ ^2^ ^,^ ^29^ ^,^ ^47^ ^,^ ^52^ ^–^ ^54^:

• Accelerate structural economic transformation through industrial policy promoting labour-intensive manufacturing to reduce dependence on low-productivity agriculture as the primary employment absorber.
• Expand quality TVET aligned with private-sector demand to address credential employment mismatch, incorporating both technical and soft skills development. ^44^ ^,^ ^45^ ^,^ ^56^
• Establish social protection floors for *NEET* youth to prevent human-capital atrophy and reduce necessity-driven informal employment. ^18^
• Mainstream gender-transformative employment policies, including anti-discrimination legislation and childcare infrastructure, and targeted programs addressing the specific barriers faced by young women, including motherhood related *NEET* risk. ^14^ ^,^ ^27^
• Strengthen sub-regional labour market integration to enlarge effective labour markets, and leverage ICT and digital platforms to improve labour market matching. ^57^
• Address energy infrastructure gaps as a complementary strategy, given evidence linking energy poverty to youth unemployment. ^58^

## Conclusions

This study provides a cross-national descriptive analysis of African youth labour markets covering 54 African Union member states in 2026, based on harmonised *ILO* modelled estimates. The continental *YUR* of 6.91%, *NEET* rate of 19.23%, Gini coefficient of 0.61, agricultural employment dominance at 45.9%, a systematic gender gap, and the educated unemployment paradox collectively define a multi-dimensional structural challenge. The regression analysis identifies agricultural employment share as the most robust structural correlate of cross-country *YUR* variation, while the educational composition of unemployment shows a consistent positive association. The descriptive patterns are consistent with all four propositions, although H _1_–H _3_ rely on unconditional mean comparisons and warrant formal testing when microdata become available. These findings highlight the need for multi-dimensional frameworks incorporating *NEET* rates, employment quality, sectoral composition, and gender, beyond *YUR* alone.

## Data Availability

The data underlying the results of this study were retrieved from the Africa Youth Employment Clock (World Data Lab), an open-access platform available at https://africayouthjobs.io, which draws on ILO harmonised modelled labour market estimates (ILOSTAT). The four underlying datasets (employed_2026.xlsx, unemployed_2026.xlsx, inactive_2026.xlsx, student_2026.xlsx) have been deposited on Zenodo: World Data Lab. Youth Labour Market Dataset Africa, 2026. https://doi.org/10.5281/zenodo.19322273. Data are available under the terms of the Creative Commons Attribution 4.0 International license (CC-BY 4.0).
