# Supplementary material for: Youth Labour Market Participation in Africa: A Cross-National Analysis of Employment, Unemployment, Sectoral Distribution, and Educational Attainment Among the 15–35 Age Cohort, 2026
Source: F1000Res. 2026 Sep 24;15:604. Originally published 2026 Apr 22. [Version 3] doi: 10.12688/f1000research.179407.3 (PMC13560831; doi:10.12688/f1000research.179407.3)
Supplement: Peer review history

# Reviewer report: Reviewer response for version 3

**DOI:** 10.5256/f1000research.209624.r524218

**Published:** 2026-09-29

**Authors:** Aryadimas Suprayitno

**Affiliations:** 1 Faculty of Economics and Business, Universitas Sumatera Utara, Medan, North Sumatra, Indonesia

Thank you for the revisions. However, there are a few points to clarify further, as the discussion is quite interesting.

 (1) In the “Statistical Analysis” section, Authors propose four models. Could Authors explain why it’s important to discuss these four models? With the addition of each new variable: M2 complementing M1, M3 complementing M2, and M4 focusing on proportions, so what research objectives or Hypothesis does each model aim to demonstrate? What kind of urgency that each Model want to show?

 (2) Delving deeper into the “Structural Transformation and Agricultural Industrialization” section, how far the possibility of implementation of a transition from traditional agriculture to industrial agriculture occurring at research location? Is this transition feasible in these countries, and what would be the potential for labor absorption in the agricultural industry if the transition is successful? Can Authors predict this based on the current pace of industrial transition in your study area: whether it is already sufficiently rapid or will take a long time, as well as the existing educational levels of young men and women and whether they would be able to be absorbed into the industrialized agricultural sector? Or can Authors present another more detailed perspective?

 (3) For the revised Policy Implications section and "Gender and labour market inequality" section, please ensure that each stakeholder is assigned one concrete step, so each stakeholder has just one detailed specific action that directly addresses what they need to do as detailed practical guideline.

 (4) Please revise the Conclusion section to include the research results and any interesting findings; do not include any statistical figures in the Conclusion section, simply highlight the key takeaways and areas requiring further evaluation.

 (5) The phrase “for country c” should include a (c) to indicate that “country” is abbreviated to “c.”

 That’s all. Hopefully will improve the quality of this paper.

Is the work clearly and accurately presented and does it cite the current literature?

Yes

If applicable, is the statistical analysis and its interpretation appropriate?

Partly

Are all the source data underlying the results available to ensure full reproducibility?

Yes

Is the study design appropriate and is the work technically sound?

No

Are the conclusions drawn adequately supported by the results?

Yes

Are sufficient details of methods and analysis provided to allow replication by others?

Partly

Reviewer Expertise:

Macroeconomics, Development Economics, Islamic Commercial and Social Finance

I confirm that I have read this submission and believe that I have an appropriate level of expertise to confirm that it is of an acceptable scientific standard, however I have significant reservations, as outlined above.

# Reviewer report: Reviewer response for version 2

**DOI:** 10.5256/f1000research.207209.r507388

**Published:** 2026-08-11

**Authors:** Gabriele Marconi

**Affiliations:** 1 Service Études et Statistiques, Agence pour le Développement de l'Emploi (ADEM), Luxembourg city, Luxembourg

I find this a very informative report on the subject of (un)employment in a region (Africa) that often is undercovered due to lack of data. The author did a good job in combining descriptive and relational statistics.

Is the work clearly and accurately presented and does it cite the current literature?

Yes

If applicable, is the statistical analysis and its interpretation appropriate?

Yes

Are all the source data underlying the results available to ensure full reproducibility?

Yes

Is the study design appropriate and is the work technically sound?

Yes

Are the conclusions drawn adequately supported by the results?

Yes

Are sufficient details of methods and analysis provided to allow replication by others?

Yes

Reviewer Expertise:

Labour economics, education economics, statistical methods

I confirm that I have read this submission and believe that I have an appropriate level of expertise to confirm that it is of an acceptable scientific standard.

# Reviewer report: Reviewer response for version 2

**DOI:** 10.5256/f1000research.207209.r508085

**Published:** 2026-08-10

**Authors:** Aryadimas Suprayitno

**Affiliations:** 1 Faculty of Economics and Business, Universitas Sumatera Utara, Medan, North Sumatra, Indonesia

Dear Authors,

 To enhance the rigor, clarity, and policy relevance of the manuscript, please address the following specific comments and revision suggestions.

 1. Regarding the consistency and granularity of your policy recommendations, the authors maintain commendable focus on "Young Women" throughout the Introduction, Results, and Discussion sections. However, in the sub-section "Gender and labour market inequality," the policy recommendations would benefit significantly from greater institutional specificity. Please specify which government ministries or departments are best positioned to spearhead these interventions to foster effective cross-ministerial collaboration. Furthermore, expand on the roles of private sector corporations and civil society communities by explaining how they can actively contribute to establishing women-friendly corporate environments and supportive community networks, and elaborate on their precise roles in driving gender equality policy interventions within industries. Then, what kind of discrimination they face in these labor markets and solutions based on stakeholders' perspective?

 2. Concerning the methodology, the study analyzes age brackets 15–19 years and 20–24 years. Given that these cohorts typically correspond to secondary and tertiary education phases during which individuals are often outside the labor force and thus categorized as non-labor force participants rather than unemployed, this methodological choice requires stronger justification. Please provide a rigorous clarification in the "Theoretical framework and literature review" sub-section, explicitly defining how youth unemployment is conceptualized and operationalized in this study, keeping in mind that a substantial portion of these age groups may still be enrolled in formal education.

 3. On structural transformation *"Discussion"* section, the manuscript correctly posits in the sub-section "Structural transformation and the agricultural trap" that transitioning from agriculture to industry can enhance economic welfare. However, framing agriculture purely as a "trap" overlooks its broader potential. Consider reframing or expanding the discussion to include the industrialization of agriculture. Rather than focusing solely on a wholesale exodus to other industrial sectors, discuss how traditional agriculture can be modernized and scaled up via industrial approaches to reframe the sector not as a structural trap, but as a high-potential area capable of industrial-scale development.

 4. Regarding the empirical findings, the statement " *This pattern whereby higher educational attainment is associated with higher measured unemployment is characteristic of transition economies and reflects the interaction of several mechanisms*" alongside the statistical finding highlights a counterintuitive relationship that requires deeper critical interpretation to avoid misleading implications. Please clarify whether this implies that higher formal education directly drives up unemployment, or if it reflects a dominance of informal labor markets where highly educated workers are reluctant to participate. Elaborate on the underlying mechanisms behind this credential–employment mismatch to ensure readers do not misinterpret the findings as an argument against pursuing higher education.

 5. In addition, the econometric model specification currently lacks sufficient transparency. Please explicitly detail the econometric equations utilized across both the Methodology and Results sections. In the Methodology section, present the formal specification of the econometric models, clearly defining all components, including the dependent variables, independent variables, parameters, intercepts, coefficients, and error terms. In the Results section, ensure that the estimated models or equations along with their specific parameter estimates, intercepts, and coefficients are clearly referenced or presented to guarantee full transparency and reproducibility.

 6. Stated energy transformation within the Policy Implication section appears abrupt, as energy poverty or energy indicators were not previously integrated into the Discussion or analyzed as part of the multidimensional poverty framework. To maintain structural and thematic coherence, please either integrate the discussion of energy poverty and energy transformation into the main analytical sections so that its inclusion in the policy implications is justified, remove the reference to energy transformation to ensure parity and fairness among multidimensional poverty indicators.

Is the work clearly and accurately presented and does it cite the current literature?

Yes

If applicable, is the statistical analysis and its interpretation appropriate?

Partly

Are all the source data underlying the results available to ensure full reproducibility?

Yes

Is the study design appropriate and is the work technically sound?

No

Are the conclusions drawn adequately supported by the results?

Yes

Are sufficient details of methods and analysis provided to allow replication by others?

Partly

Reviewer Expertise:

Macroeconomics, Development Economics, Islamic Commercial and Social Finance

I confirm that I have read this submission and believe that I have an appropriate level of expertise to confirm that it is of an acceptable scientific standard, however I have significant reservations, as outlined above.

# Response

**Published:** 2026-08-29

**Authors:** Said Maizzou

**Affiliations:** Economics and Management, Universite Hassan 1er, Settat, Casablanca-Settat, Morocco

Dear Reviewer,

 We sincerely thank you for the detailed and constructive comments. We have carefully revised the manuscript to improve its methodological clarity, policy relevance, interpretation, and reproducibility. Our responses are provided below.

**Comment 1: Gender inequality and institutional responsibilities**

 We agree that the policy recommendations required greater institutional specificity. The revised manuscript now identifies the responsibilities of:

 - Ministries of Labour and Employment: enforcement of equal-pay, anti-discrimination, maternity-protection, and workplace-safety legislation.

 - Ministries responsible for Gender or Women’s Affairs: gender-responsive budgeting, monitoring, and complaints mechanisms.

 - Ministries of Education and Higher Education: curriculum alignment, apprenticeships, and career services.

 - Ministries of Industry, Agriculture, Digital Economy, and Small-Business Development: access to finance, productive assets, procurement, technology, and value chains.

 We also expanded the roles of private corporations, civil-society organisations, women’s associations, trade unions, and community leaders. The manuscript now discusses discrimination in recruitment, remuneration, promotion, occupational segregation, pregnancy and motherhood, sexual harassment, and access to finance. It also proposes practical measures such as pay audits, transparent recruitment, childcare partnerships, safe workplaces, mentoring, legal support, and community-based referral networks.

 These revisions were made in the sections “Gender and labour market inequality” and “Policy implications.”

**Comment 2: Justification of the 15–19 and 20–24 age groups**

 We agree that many individuals in these age groups may still be enrolled in education. We therefore added a clarification to the “Theoretical framework and literature review” section.

 Youth unemployment is now explicitly defined according to the ILO labour-force framework. A young person is classified as unemployed only when they are not employed, actively seeking work, and available to start work. The unemployment rate is calculated using the labour force as the denominator, rather than the total youth population.

 Students are not classified as unemployed merely because they do not work. They are included in the unemployment measure only when they satisfy the activity and availability criteria. The manuscript also clarifies that the estimates for the 15–19 and 20–24 cohorts represent labour-market outcomes among active participants and should not be interpreted as evidence that education causes unemployment.

**Comment 3: Structural transformation and agriculture**

 We agree that describing agriculture exclusively as an “agricultural trap” was too narrow. The section has been renamed “Structural transformation and agricultural industrialisation.”

 The revised discussion now presents agriculture as a potential high-productivity sector. It addresses irrigation, climate-resilient inputs, mechanisation, storage, cold chains, agro-processing, digital extension services, rural finance, transport infrastructure, cooperatives, and producer organisations.

 The revised manuscript argues that structural transformation should not require a wholesale exit from agriculture. Instead, agricultural upgrading should be combined with industrialisation in manufacturing and services, allowing young people who remain in rural areas to benefit from formalisation, value addition, and higher-productivity employment.

**Comment 4: Interpretation of educated unemployment**

 We agree that the original wording could be misinterpreted as suggesting that education directly causes unemployment. The interpretation has been revised.

 The manuscript now clearly states that the positive association between education and measured unemployment does not establish a causal effect of formal education. Several mechanisms may explain the finding:

 - More educated youth generally have higher labour-force participation.

 - They may search longer for jobs corresponding to their qualifications.

 - They may be less likely to accept poorly paid, insecure, or informal employment.

 - Educational expansion may have outpaced the creation of skilled jobs.

 - Curriculum content may not correspond sufficiently to employer demand.

 We also clarify that the available aggregate data do not permit a definitive test of credential–employment mismatch. The results should therefore not be interpreted as an argument against higher education. Rather, they support stronger school-to-work transitions, curriculum reform, skills development, and skilled job creation.

**Comment 5: Econometric model transparency**

 We agree with this recommendation. The “Statistical analysis” section now provides the formal specifications for Models 1–4, including:

 - The dependent variable.

 - All independent variables.

 - Intercepts.

 - Slope coefficients.

 - Error terms.

 - Definitions of the North Africa dummy, gender gap, agricultural employment share, and educated-unemployed share.

 The revised manuscript also clarifies that Model 5 is a log-transformed robustness specification rather than one of the four primary nested models.

 The Results section now reports the estimated intercepts and coefficients corresponding to each model, with standard errors and significance levels presented in Table 4. This improves transparency and facilitates replication.

**Comment 6: Energy transformation**

 We agree that the reference to energy transformation was insufficiently integrated into the analytical framework. Since energy poverty was not directly analysed as part of the multidimensional poverty framework, we removed the energy-related recommendation from the “Policy implications” section.

 This revision ensures consistency between the variables analysed in the manuscript and the policy recommendations presented.

 We appreciate the reviewer’s positive assessment regarding the clarity of presentation, citation of current literature, source-data availability, and support for the conclusions. The revisions described above specifically address the concerns regarding technical soundness, methodological detail, and statistical interpretation.

 We thank the reviewer again for helping us improve the manuscript.

# Reviewer report: Reviewer response for version 1

**DOI:** 10.5256/f1000research.197913.r489565

**Published:** 2026-07-10

**Authors:** Gabriele Marconi

**Affiliations:** 1 Service Études et Statistiques, Agence pour le Développement de l'Emploi (ADEM), Luxembourg city, Luxembourg

-> H1 is " *YUR* is significantly higher in North Africa than in sub-Saharan Africa, reflecting formal-sector scarcity and widespread informal agricultural employment". The first part is a hypothesis that is tested in the paper " *YUR* is significantly higher in North Africa than in sub-Saharan Africa", but the second "reflecting formal-sector scarcity and widespread informal agricultural employment" is not in itself a testable hypothesis (more a potential explanation) so it should not be mentioned here.

 -> Similarly, in H3, the testable hypothesis is "Youth with upper secondary and tertiary qualifications account for a disproportionate share of unemploy-ment relative to their population weight"; "credential–employment mismatch" is just a possible explanation and should not be mentioned here.

 -> Equation (1): YUR should be, I guess, "U / (E+U)", not "(U E) / (E+U)"

 -> Equation (3): This is not a NEET rate, unless you subtract people in unemployment or training

 -> The resolution of the figures is very low, which makes them very difficult to read

 -> About the conclusions in Section 3.3 (age-group differentials), I believe that the unemployment rate is not a good indicator of labour market outcomes during the phase of transition between study and work. The reason is that students can be both studying and looking for work, and their response to survey questions on labour participation are difficult to predict. This should at least be acknowledged here, especially given that the "inadequacy of YUR alone" is one of your conclusions. I developed this point in a more structured way in a previous study, ( ***Marconi et al., 2016***) **.**

 -> Concerning Section 3.4, I am a bit surprised that the gender comparison, does not include the NEET rate. In an in-depth comparative analysis of NEET rates across developed countries, my co-authors and I found that motherhood, in particular, was one of the biggest drivers ( ***Kevelson M et al. 2020***). 

 -> In Section 3.6 you state "This *educated unemployment paradox* reflects structural mismatches between curricula and private-sector demand". That can be, but as you pointed out previously, the unemployment rate is strongly affacted by the activitiy rate. So your result could be, at least in part, driven by higher activity rates among more educated people.

Is the work clearly and accurately presented and does it cite the current literature?

Yes

If applicable, is the statistical analysis and its interpretation appropriate?

Yes

Are all the source data underlying the results available to ensure full reproducibility?

Yes

Is the study design appropriate and is the work technically sound?

Yes

Are the conclusions drawn adequately supported by the results?

Yes

Are sufficient details of methods and analysis provided to allow replication by others?

Yes

Reviewer Expertise:

Labour economics, education economics, statistical methods

I confirm that I have read this submission and believe that I have an appropriate level of expertise to confirm that it is of an acceptable scientific standard, however I have significant reservations, as outlined above.

## References

[1] : Age effects in Okun’s law with different indicators of unemployment. *Applied Economics Letters* .2016;23(8) : 10.1080/13504851.2015.1090540580-583 10.1080/13504851.2015.1090540

[2] : College Educated Yet Disconnected: Exploring Disconnection From Education and Employment in OECD Countries, With a Comparative Focus on the U.S. *ETS Research Report Series* .2020;2020(1) : 10.1002/ets2.123051-29 10.1002/ets2.12305

# Response

**Published:** 2026-07-23

**Authors:** Said Maizzou

**Affiliations:** Economics and Management, Universite Hassan 1er, Settat, Casablanca-Settat, Morocco

Response to Reviewer 3 — Gabriele Marconi

*(Service des Etudes et Statistiques, Agence pour le D´eveloppement de l’Emploi (ADEM), Luxem-´ bourg)*

 We thank Reviewer 3 for the precise technical corrections and the pertinent bibliographic references.

**Comment 1: “H**_1_ **is ‘YUR is significantly higher in North Africa than in sub-Saharan Africa’ — the first part is a hypothesis that is tested, but the second ‘reflecting formal-sector scarcity and widespread informal agricultural employment’ is not in itself a testable hypothesis.”**

**Response: **We agree. H _1 _has been revised to remove the interpretive clause. The revised H _1 _reads:

**H**_1_ **:** *YUR*is significantly higher in North Africa than in sub-Saharan Africa.

 The explanatory interpretation (formal-sector scarcity, informal agricultural employment) is now discussed in the Results and Discussion sections, where the finding is interpreted in light of sectoral composition differences.

**Comment 2: “Similarly, in H**_3_ **, ‘credential–employment mismatch’ is just a possible explanation and should not be mentioned here.”**

**Response: **We agree. H _3 _has been revised to:

**H**_3_ **: **Youth with upper secondary and tertiary qualifications account for a disproportionate share of unemployment relative to their population weight.

 The term “credential–employment mismatch” is now used only in the Discussion when interpreting the finding, not in the hypothesis itself.

**Comment 3: “Equation (1): YUR should be, I guess, U / (E+U), not (U E) / (E+U).”**

**Response: **The YUR equation in the manuscript is already correctly specified as:

 Unemployed

 YUR = × 100 Employed + Unemployed

 This is consistent with the standard ILO definition. The reviewer may have been referring to an earlier version of the manuscript. We have verified that the current version is correct.

**Comment 4: “Equation (3): This is not a NEET rate, unless you subtract people in unemployment or training.”**

**Response: **The NEET equation is correctly specified.

 We have added a clarifying note immediately following the equation:

 “Inactive” refers to economically inactive youth who are neither employed, unemployed, nor in education or training, consistent with the standard ILO NEET definition (ILO, 2023). Students enrolled in education or training are excluded from both the numerator and the “Inactive” category.

 This makes explicit that the denominator includes all youth (including students), while the numerator counts only those who are neither employed, nor unemployed, nor in education/training.

**Comment 5: “The resolution of the figures is very low.”**

**Response: **As noted in our response to Reviewer 1, we have added a statement in the Methods section specifying a minimum resolution of 300 dpi for all figures, with a minimum font size of 8pt for all text elements.

**Comment 6: “The unemployment rate is not a good indicator of labour market outcomes during the phase of transition between study and work. This should at least be acknowledged, especially given that the ‘inadequacy of YUR alone’ is one of your conclusions. I developed this point in a more structured way in a previous study (Marconi et al., 2016).”**

**Response: **We have added this important caveat in Section 3.3 (Age-Group Differentials):

 “The unemployment rate may not be an informative indicator of labour market outcomes during the transition from education to work, because students can be simultaneously enrolled in education and actively seeking employment; their responses to survey questions on labour force participation are difficult to interpret (Marconi et al., 2016). The relatively high YUR observed in the 15–19 group (9.41%) should therefore be interpreted with caution, as it partly reflects high labour force attachment among students rather than purely involuntary joblessness.”

 We now cite Marconi et al. (2016) in both the Results and Discussion sections to support this methodological caveat.

**Comment 7: “I am a bit surprised that the gender comparison does not include the NEET rate. In a comparative analysis, I found that motherhood was one of the biggest drivers (Kevelson et al., 2020).”**

**Response: **We have expanded the gender analysis in Section 3.4 to include the *NEET*rate:

 “Gender disparities are further visible in the *NEET*rate, where female youth recorded substantially higher disengagement than male youth, reflecting the combined effects of lower participation, higher unemployment, and greater exclusion from education and training among young women (Kevelson et al., 2020). Motherhood has been identified as one of the strongest predictors of *NEET*status in cross-country analyses, suggesting that fertility-related barriers are a critical driver of gender disparities in youth labour market attachment (Kevelson et al., 2020).”

 The Discussion (Section 4.4) further develops this point, noting that the labour market’s capacity to absorb workers varies according to workforce characteristics, not only gender per se.

**Comment 8: “Your result could be, at least in part, driven by higher activity rates among more educated people.”**

**Response: **We have thoroughly addressed this confound in both the Results (Section 3.5) and Discussion (Section 4.3). In the Results, we note:

 “This pattern may be partly driven by higher labour force participation rates among more educated youth: because the unemployment rate is conditional on activity, higher search intensity and stronger formal-sector attachment among educated youth mechanically expand the pool of individuals who can be counted as unemployed (Marconi et al., 2016).”

 In the Discussion, we expand this into a three-part theoretical explanation (credential inflation, activity rate confound, enrolment–employment gap) and acknowledge that a definitive test requires data not available in the aggregate *ILO*dataset.

# Reviewer report: Reviewer response for version 1

**DOI:** 10.5256/f1000research.197913.r492252

**Published:** 2026-07-08

**Authors:** Włodzimierz Kołodziejczak

**Affiliations:** 1 Poznan University of Life Sciences, Poznan, Poland

This article examines the situation of young people in the African labour market. It addresses the issues of structural imbalances, gender inequality and skills mismatches. The YUR, LFPR and NEET indicators were used. The source data are from the International Labour Organisation.  The authors highlight heterogeneity, informal structures, gender inequality and social protection.

 The article accurately assesses the situation, whilst taking the demographic context into account. The analysis is generally sound and provides valuable insights. However, I would not overuse the terms ‘hypothesis’ and ‘hypothesis testing’, as in their current form they relate to observations of the actual state of affairs rather than the search for answers to research questions, for example using quantitative methods. Nevertheless, I do not recommend building econometric models. The high heterogeneity of the African economy, frequent economic, political and climatic shocks, and the varying and time-dependent resilience to these shocks make it very difficult to construct a reliable – rather than merely formally sound – model.

 In my view, the authors’ calculations are sufficient, and the key to success lies in their interpretation. In its current form, the article reads more like a research report than an academic text. It is therefore essential to ground the analysis in economic theory.

 Firstly, a review of the literature on labour market theory should be conducted, the most important findings of other authors’ research should be described, and the authors’ own analysis should be situated within this framework. Following the presentation of the results, a discussion of these findings is also required, based on the literature and economic theory, which can lead to the formulation of conclusions and, subsequently, recommendations for economic policy. The current version of the article contains certain recommendations (section 4.7) and conclusions set out further on. This is not the correct sequence, as the research problem must first be resolved, and only on this basis can recommendations be formulated. These rather general recommendations have not been supported by an analysis of the causes of the identified phenomena. In particular, the negative correlation between educational attainment and employment – typical of transition economies – requires explanation in the discussion.

 Another important issue is the formally worse situation of women in the labour market. Here, attributing this solely to discrimination is insufficient, as the labour market’s capacity naturally varies according to the characteristics of the workforce, not just gender.  In my view, the analysis should also take into account the potential impact of migration. Africa’s rapid population growth will fuel migratory pressure. As might be expected, young people will constitute the largest group of emigrants from Africa.

 From a technical perspective, the clarity of the figures needs to be improved, as their low resolution makes it impossible to make out the content. I suggest not dividing the text into too many headings, particularly when discussing the results. A better approach is to maintain a logical structure of the text, following the principle of ‘from the general to the specific’, so that the reader gains an understanding not only of the overall picture but also of the nature and context of the phenomena under study.

Is the work clearly and accurately presented and does it cite the current literature?

Partly

If applicable, is the statistical analysis and its interpretation appropriate?

Not applicable

Are all the source data underlying the results available to ensure full reproducibility?

Yes

Is the study design appropriate and is the work technically sound?

Partly

Are the conclusions drawn adequately supported by the results?

Partly

Are sufficient details of methods and analysis provided to allow replication by others?

Yes

Reviewer Expertise:

Labour market, unemployment, labour productivity, sustainable development

I confirm that I have read this submission and believe that I have an appropriate level of expertise to confirm that it is of an acceptable scientific standard, however I have significant reservations, as outlined above.

# Response

**Published:** 2026-07-23

**Authors:** Said Maizzou

**Affiliations:** Economics and Management, Universite Hassan 1er, Settat, Casablanca-Settat, Morocco

Response to Reviewer 2 — W lodzimierz Kol odziejczak

*(Poznan University of Life Sciences, Poznan, Poland)*

 We thank Reviewer 2 for the thorough and insightful commentary. The recommendations to ground the analysis in economic theory and to restructure the article have significantly improved the manuscript.

**Comment 1: “I would not overuse the terms ‘hypothesis’ and ‘hypothesis testing’, as in their current form they relate to observations of the actual state of affairs rather than the search for answers to research questions.”**

**Response: **We have revised the terminology throughout the manuscript. In the Hypotheses subsection, we now state that the study “formulates four testable propositions grounded in descriptive comparisons” rather than “tests four hypotheses.” In the Discussion and Conclusions, we refer to “findings broadly consistent with all four propositions” rather than “confirming all four hypotheses.” In the Results, we use “consistent with H _1_” rather than “supports H _1_.” These changes better reflect the descriptive–correlational nature of our analysis and avoid implying inferential hypothesis testing where unconditional mean comparisons are presented.

**Comment 2: “The article reads more like a research report than an academic text. It is essential to ground the analysis in economic theory. A review of the literature on labour market theory should be conducted, the most important findings of other authors’ research should be described, and the authors’ own analysis should be situated within this framework.”**

**Response: **We have added a new subsection titled “ **Theoretical Framework and Literature Review**” in the Introduction (revised Section), which situates the study within the following theoretical traditions:

- **Structural transformation theory **(McMillan et al., 2023): economic development entails progressive reallocation of labour from low-productivity agriculture to higherproductivity manufacturing and services.
- **The Harris–Todaro model **(Harris & Todaro, 1970): segmented labour markets generate unemployment as an equilibrium outcome when formal-sector wage premiums attract job-seekers.
- **Signalling and screening theories of education **(Spence, 1973; Stiglitz, 1975; Dore, 1976): credential inflation occurs when educational expansion outpaces high-skilled job creation.
- **Search-and-matching models **(Pissarides, 2000): frictional and structural mismatches between worker skills and employer requirements generate equilibrium unemployment.

The Discussion section has been entirely restructured to interpret all findings within this theoretical framework. For example, the negative coefficient on agricultural share is now interpreted through the Harris–Todaro model of segmented labour markets, and the educated unemployment paradox is discussed in terms of signalling theory and the “diploma disease” phenomenon (Dore, 1976).

**Comment 3: “The negative correlation between educational attainment and employment — typical of transition economies — requires explanation in the discussion.”**

**Response: **We have considerably expanded the discussion of the educated unemployment paradox (revised Section 4.3). We now present three complementary explanatory mechanisms:

1. **Credential inflation **(Spence, 1973; Stiglitz, 1975; Dore, 1976): when educational credentials become widely accessible without a corresponding expansion of skilled employment, the informational content of degrees diminishes.
2. **Activity rate confound **(Marconi et al., 2016): higher educational attainment is associated with higher labour force participation, which mechanically increases the pool of individuals who can be counted as unemployed. The observed pattern may therefore reflect higher search intensity among educated youth rather than purely skills mismatch.
3. **Enrolment–employment gap **(Njifen, 2024): rapid expansion of tertiary enrolment in Africa has outpaced the growth of high-skilled employment, creating a structural surplus of credentialed workers.

We acknowledge that a definitive test requires comparing the educational distribution of the unemployed with that of the employed labour force, which is not possible with the aggregate data available.

**Comment 4: “Attributing this solely to discrimination is insufficient, as the labour market’s capacity naturally varies according to the characteristics of the workforce, not just gender.”**

**Response: **We have revised the gender discussion (Section 4.4) to acknowledge that workforce characteristics—not only gender per se—explain labour market disparities. We now discuss:

- Differential sectoral composition of female and male labour supply (women concentrated in services vs. men in industry).
- Occupational segregation driven by social norms and care responsibilities.
- Motherhood as a key mediating factor, citing Kevelson et al. (2020), who identify it as one of the strongest predictors of *NEET*status in cross-country analysis.

**Comment 5: “The analysis should also take into account the potential impact of migration.”**

**Response: **We have added a new subsection on “ **Informal Economy, Necessity Employment, and Migration**” (Section 4.5), which discusses:

- Africa’s rapid population growth and its implications for migratory pressures, with youth constituting the largest cohort of potential emigrants.
- The Harris–Todaro framework for understanding migration decisions driven by expected income differentials.
- The brain-drain mechanism whereby emigration depletes human capital (Clemens et al., 2022).
- The limitation that the current analysis does not capture migration flows, which is flagged as an important direction for future research.

**Comment 6: “The current version of the article contains certain recommendations (section 4.7) and conclusions set out further on. This is not the correct sequence.”**

**Response: **We have restructured the final sections of the manuscript to follow the correct logical sequence: **Discussion **→ **Conclusions **→ **Policy Implications**. The Policy Implications are now presented as Section 6, following the Conclusions (Section 5), and are explicitly framed as derived from the preceding analysis and discussion.

**Comment 7: “I suggest not dividing the text into too many headings, particularly when discussing the results.”**

**Response: **We have corrected the section numbering in the Results (removed the gap at section 3.5) and ensured sequential numbering (3.1–3.7). We have maintained the current subsection structure because each addresses a distinct research question or hypothesis, which aids readability. However, we have streamlined subheading titles where possible.

# Reviewer report: Reviewer response for version 1

**DOI:** 10.5256/f1000research.197913.r477644

**Published:** 2026-05-20

**Authors:** Sudipa Sarkar

**Affiliations:** 1 Institute of Public Policy, National Law School of India University, Bangalore, Karnataka, India

Comments to the author(s) on the paper “Youth Labour Market Participation in Africa: A Cross-National Analysis of Employment, Unemployment, Sectoral Distribution, and Educational Attainment Among the 15–35 Age Cohort, 2026”

 The manuscript investigates youth employment participation in Africa using cross country data. The study undertakes a descriptive analysis of the labour force data in the African countries. The analysis is comprehensive and presents an overview of the labour market indicators. However, the paper in its current form reads as a comprehensive report and lack sufficient depth and analytical rigour to be considered as a research article. I would suggest the authors to undertake some regression analysis to go one step further in analysing the indicators associated with the youth employment rate in a single model. This will allow the authors in analysing the partial correlation between the employment participation and other demographic and human capital indicators included in the descriptive analysis. 

 I would also suggest the authors to make the figures readable. In its current form, the figures are not clearly visible and the numbers are not readable.

Is the work clearly and accurately presented and does it cite the current literature?

Yes

If applicable, is the statistical analysis and its interpretation appropriate?

Partly

Are all the source data underlying the results available to ensure full reproducibility?

Yes

Is the study design appropriate and is the work technically sound?

Partly

Are the conclusions drawn adequately supported by the results?

Partly

Are sufficient details of methods and analysis provided to allow replication by others?

Yes

Reviewer Expertise:

Future of work, skills, social inequality

I confirm that I have read this submission and believe that I have an appropriate level of expertise to confirm that it is of an acceptable scientific standard, however I have significant reservations, as outlined above.

# Response

**Published:** 2026-05-21

**Authors:** Said Maizzou

**Affiliations:** Economics and Management, Universite Hassan 1er, Settat, Casablanca-Settat, Morocco

We sincerely thank the reviewer for the careful reading of our manuscript and for the constructive and insightful comments.

 We are encouraged by the reviewer’s positive assessment regarding the relevance of the topic, the comprehensiveness of the labour market indicators, and the clarity of the methodological documentation and data availability.

 We fully acknowledge the reviewer’s important observation that the manuscript, in its current form, is closer to a descriptive analytical report than to a fully developed empirical research article. In response, we will substantially strengthen the analytical component of the study.

 More specifically, we will incorporate multivariate regression analyses to examine the relationship between youth employment participation and key demographic, educational, and labour-market variables across African countries. This additional analysis will allow us to move beyond descriptive comparisons and assess the partial correlations between youth employment outcomes and explanatory indicators within a unified analytical framework.

 We also appreciate the reviewer’s recommendation concerning the readability of the figures. In the revised version, all figures will be redesigned with improved resolution, formatting, and labeling to ensure clarity and readability.

 We are grateful for these valuable suggestions, which will significantly improve the quality, analytical depth, and overall contribution of the manuscript.

# Response

**Published:** 2026-07-23

**Authors:** Said Maizzou

**Affiliations:** Economics and Management, Universite Hassan 1er, Settat, Casablanca-Settat, Morocco

Response to Reviewer 1 — Sudipa Sarkar

*(Institute of Public Policy, National Law School of India University, Bangalore, India) *We thank Reviewer 1 for the positive assessment of the data quality and methodology, and for the constructive suggestions to strengthen the analytical depth of the paper.

**Comment 1: “The paper reads as a comprehensive report and lacks sufficient depth and analytical rigour. I would suggest the authors to undertake some regression analysis.”**

**Response: **We fully agree that a regression analysis adds analytical depth beyond descriptive statistics. We have now incorporated four nested ordinary least squares (OLS) regression models with the *YUR*as the dependent variable (Section 3.7, revised Table **??**). The models use only conceptually independent predictors—agricultural employment share, a North Africa regional dummy, the gender gap in *YUR*, and the share of unemployed with upper-secondary or tertiary education—because *NEET*rate and *LFPR*are mechanically related to *YUR*and would render the regression tautological. The sectoral model (M _1_) alone explains 33.1% of cross-country

*YUR*variance ( *R*_adj_^2 ^= 0 *.*319; agriculture share *β *= −0 *.*255, *p < *0 *.*001), and the full model (M _3_) reaches an adjusted *R* ^2 ^of 0.366. These results identify agricultural employment share as the most robust structural correlate of cross-country *YUR*variation, while the educational composition of unemployment shows a consistent positive association. The abstract, Methods, Results, and Discussion sections have all been updated to present these findings.

**Comment 2: “The figures are not clearly visible and the numbers are not readable.”**

**Response: **We have added an explicit statement in the Statistical Analysis subsection (Methods) specifying that all figures are produced at a minimum resolution of 300 dpi to ensure legibility of numerical labels and colour coding at print size. We have also revised the Python code used to generate the figures to ensure that all text elements (axis labels, tick labels, annotations) are rendered at a minimum font size of 8pt, and that colour palettes are colourblind-safe.
